# Impact of vaccination on the COVID-19 pandemic in U.S. states

**DOI:** 10.1038/s41598-022-05498-z

**Published:** 2022-01-28

**Authors:** Xiao Chen, Hanwei Huang, Jiandong Ju, Ruoyan Sun, Jialiang Zhang

**Affiliations:** 1 School of International Trade and Economics, University of International Business and Economics, Beijing, China; 2 Department of Economics and Finance, City University of Hong Kong, Hong Kong, China; 3 Centre for Economic Performance, London School of Economics, London, United Kingdom; 4 PBC School of Finance, Tsinghua University, Beijing, China; 5 Department of Health Care Organization and Policy, School of Public Health, University of Alabama at Birmingham, Birmingham, USA

**Keywords:** Viral infection, Viral infection

## Abstract

Governments worldwide are implementing mass vaccination programs in an effort to end the novel coronavirus (COVID-19) pandemic. Here, we evaluated the effectiveness of the COVID-19 vaccination program in its early stage and predicted the path to herd immunity in the U.S. By early March 2021, we estimated that vaccination reduced the total number of new cases by 4.4 million (from 33.0 to 28.6 million), prevented approximately 0.12 million hospitalizations (from 0.89 to 0.78 million), and decreased the population infection rate by 1.34 percentage points (from 10.10 to 8.76%). We built a Susceptible-Infected-Recovered (SIR) model with vaccination to predict herd immunity, following the trends from the early-stage vaccination program. Herd immunity could be achieved earlier with a faster vaccination pace, lower vaccine hesitancy, and higher vaccine effectiveness. The Delta variant has substantially postponed the predicted herd immunity date, through a combination of reduced vaccine effectiveness, lowered recovery rate, and increased infection and death rates. These findings improve our understanding of the COVID-19 vaccination and can inform future public health policies.

## Introduction

The novel coronavirus (COVID-19) pandemic has had a devastating impact on health and well-being, with more than 131 million cases and 2.8 million deaths across more than 200 countries^1^ as of early April 2021. Despite various regional and national non-pharmaceutical interventions^2–4^ such as travel restrictions, social distancing measures, stay-at-home orders, and lockdowns, many countries continue to struggle with the growth of COVID-19 cases. It is obvious that a successful COVID-19 vaccination program is needed to end the pandemic and allow a return to normal life^5,6^.

By the end of February 2021, two COVID-19 vaccines had been approved in the U.S.: BNT162b2 (Pfizer/BioNTech) and mRNA-1273 (Moderna)^7^. In two large randomized controlled trials (RCTs), the Pfizer vaccine exhibited an efficacy of 95% (95% confidence interval [CI], 90.3%–97.6%)^8^ in preventing COVID-19, and the Moderna vaccine showed an efficacy of 94.1% (95% CI, 89.3%–96.8%)^9^. Both are mRNA vaccines that require two doses to complete vaccination and received emergency use authorization by the U.S. Food and Drug Administration in December 2020^10^. Mass vaccination campaigns with these two vaccines have since begun. By early March 2021, more than 121 million doses had been administered across the U.S., with over 43 million individuals (~ 13% of the population) fully vaccinated with two doses^11^.

Although the efficacies of these two vaccines were shown to be high in RCTs, there is limited information on their potential population-level impact on the COVID-19 pandemic. One peer-reviewed study that estimated vaccine effectiveness used data from nationwide mass vaccination in Israel and reported the effectiveness of the Pfizer vaccine to be 46% (95% CI, 40%–51%) after the first dose and 92% (95% CI, 88%–95%) after the second dose for documented infection^12^. Another study that examined the effectiveness of the Pfizer vaccine among U.S. residents in skilled nursing facilities reported an estimation of 63% (95% CI, 33%–79%) after the first dose^13^.

In this study, we employed well-established reduced-form econometric techniques^14^, commonly used to evaluate the effects of policies or events^15,16^, to assess the impact of early-stage vaccination during the ongoing outbreak using data from all 50 U.S. states and the District of Columbia (DC). Although the allocation of vaccines is roughly proportional to state population (Extended Data Fig. 1a), the actual proportion of the vaccinated population differs significantly across states over time (Extended Data Fig. 1b), which provides the key variation to identify the impact of vaccination. Effectively, the observations from each region in the weeks before the vaccination program served as the “control” for the observations after the vaccination program began (“treatment”), with variations in the vaccination rates leading to changes in the “treatment intensity.” By comparing the outcomes across states before and after the initiation of vaccination programs, we evaluated the impact of vaccination on the COVID-19 pandemic.

**Figure 1:**
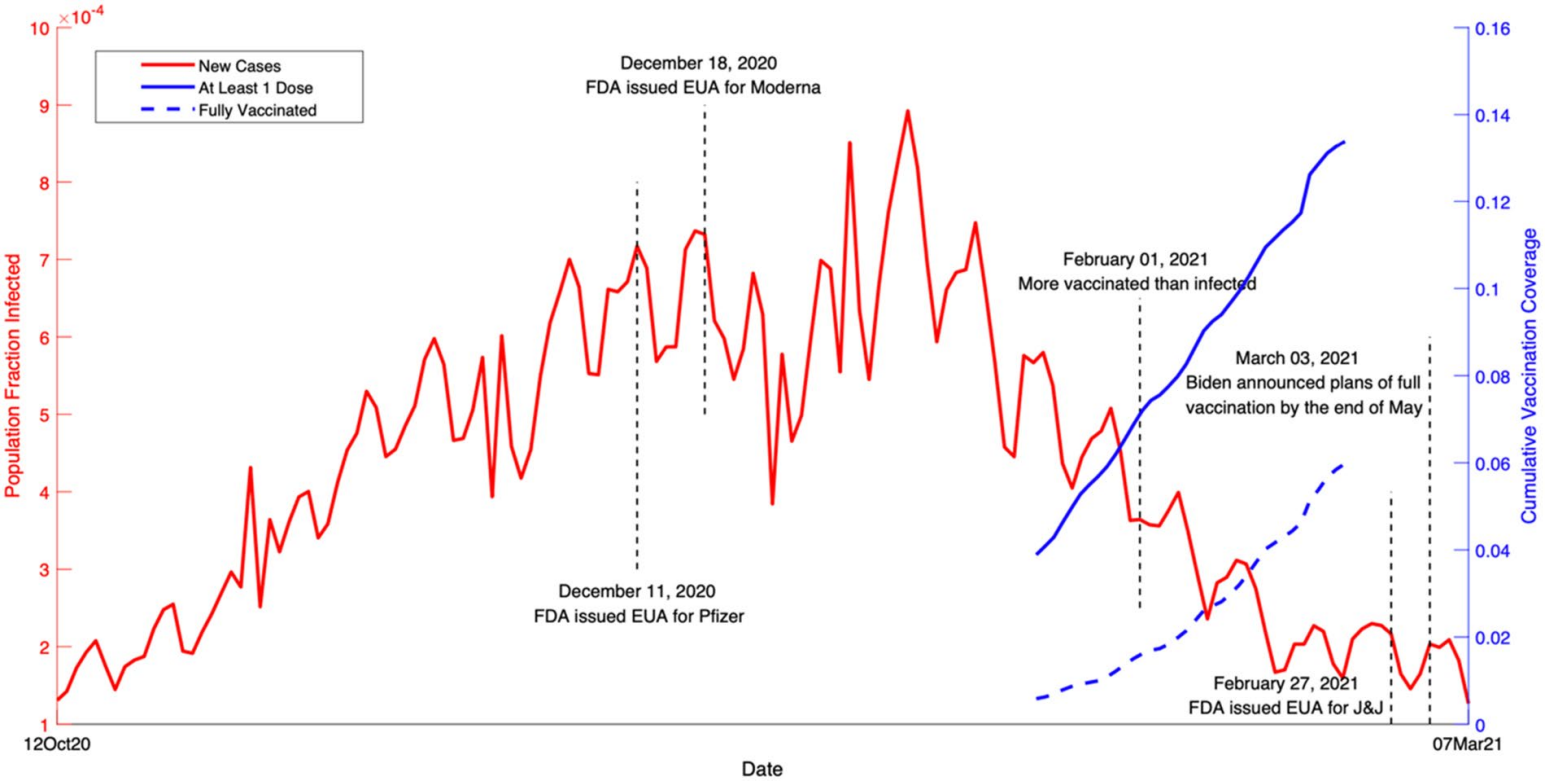
COVID-19 events and vaccination timeline in the U.S. from 12 October 2020 to 7 March 2021. The red curve is the fraction of population infected over time (left y-axis). The solid blue curve is the cumulative vaccination coverage in the population with at least one dose of vaccine (right y-axis). The dashed blue curve is the cumulative vaccination coverage of fully vaccinated individuals in the population (right y-axis).

## Results

### Study design

**Figure 2:**
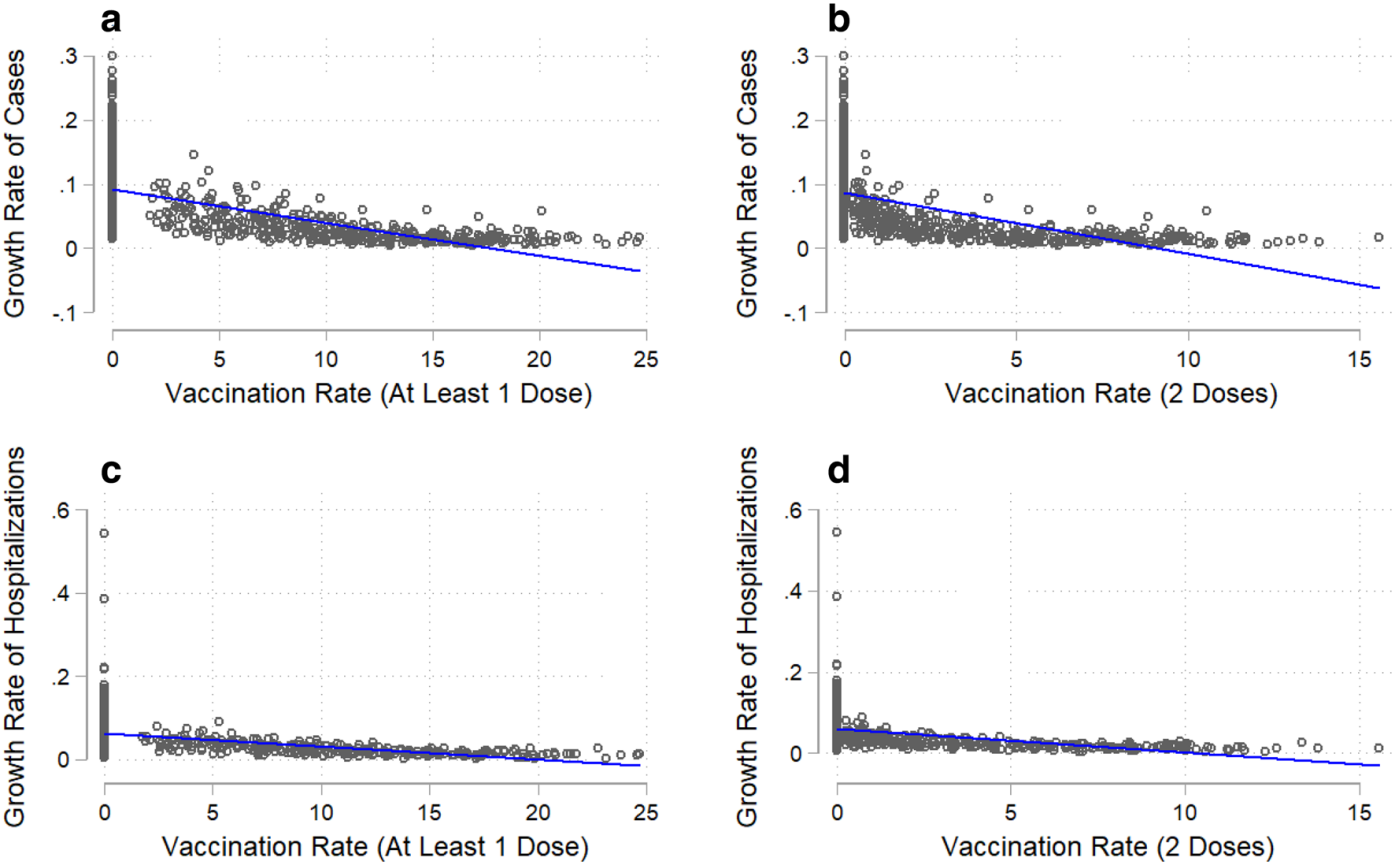
COVID-19 infections (total cases and hospitalizations) and vaccination rate. Vaccination rate is the number of individuals vaccinated per hundred. The solid line in each figure is a fitted linear curve between the growth rate of total cases/hospitalizations and vaccination rate. (**a**), Association between the growth rate of total cases and at least 1 dose of vaccination (coefficient = − 0.006, R^2^ = 35.3%). (**b**) Association between the growth rate of total cases and 2 doses of vaccination (coefficient = − 0.013, R^2^ = 28.6%). (**c**) Association between the growth rate of total hospitalizations and at least 1 dose of vaccination (coefficient = − 0.003, R^2^ = 20.8%). (**d**), Association between the growth rate of total hospitalizations and 2 doses of vaccination (coefficient = − 0.007, R^2^ = 16.6%).

We collected state-level daily infection and hospitalization data in the U.S. from 12 October 2020 to 7 March 2021. Figure 1 shows a timeline of COVID-19 developments during this period, including important events and vaccination timeline. We aggregated the data to a weekly level in our baseline estimation given the observed weekly cycle^17,18^ (see Extended Data Table 3 for results using daily data). The dependent variables used to assess the impact of vaccination on the pandemic are the growth rates of total cases and hospitalizations. Our key independent variables are vaccination rates, including the total number of vaccine doses administered per 100 people (at least one dose) and the total number of second doses administered per 100 people. Without any control variables, Fig. 2 shows the negative correlation between the vaccination rate and the growth rates of total cases and hospitalizations.

We analyzed data in the U.S. from 12 October 2020 to 7 March 2021 for three main reasons. First, we selected similar number of weeks for the pre-treatment and post-treatment periods to balance the sample in our baseline results. Second, two important variables, growth of total hospitalizations and testing, are only available till early March 2021. Third, the Delta variant started to spread since March 2021 and became the dominant strain in the U.S. by July 2021. The presence of the Delta variant has significantly changed vaccine effectiveness, along with infection rate and recovery rate^19^. Thus, we chose to examine the effect of early-stage vaccination (till 7 March 2021) in our main text, and leave the analysis of extended data up to 17 November 2021 in the Discussion.

To make the individual states as comparable as possible, we first accounted for observable factors associated with the COVID-19 pandemic based on previous studies (see Extended Data Table 1). These time-varying control variables included non-pharmaceutical interventions^5–7^, election rallies^20,21^ and anti-racism protests^22^ that involved mass gatherings, and climate measures of snow depth and temperature^23^. To address the concern that changes in the number of total cases reflect the testing capacity of each state^24^, we also controlled for each state’s testing capacity. As the proportion of susceptible individuals declines, the infection rate may slow; therefore, we included the share of susceptible individuals in the regressions. We estimated the dependent variables of COVID-19 cases and hospitalizations with a one-week lag to account for the latency period of infection. Finally, we added state fixed effects and time fixed effects to capture spatial and temporal invariants to alleviate omitted-variable bias.

### Impact of vaccination

Our data show that the national average weekly growth rate of total cases was 7% (s.e.m. = 5%) between 12 October 2020 and 7 March 2021. At the individual state level, the average growth rate was highest in Vermont (11%) and lowest in Hawaii (4%). The average growth rate of total hospitalizations across the 35 states that reported hospitalization data was 5% (s.e.m. = 4%); the highest growth rate was seen in Montana (8%) and the lowest in New Hampshire (2%).

**Figure 3:**
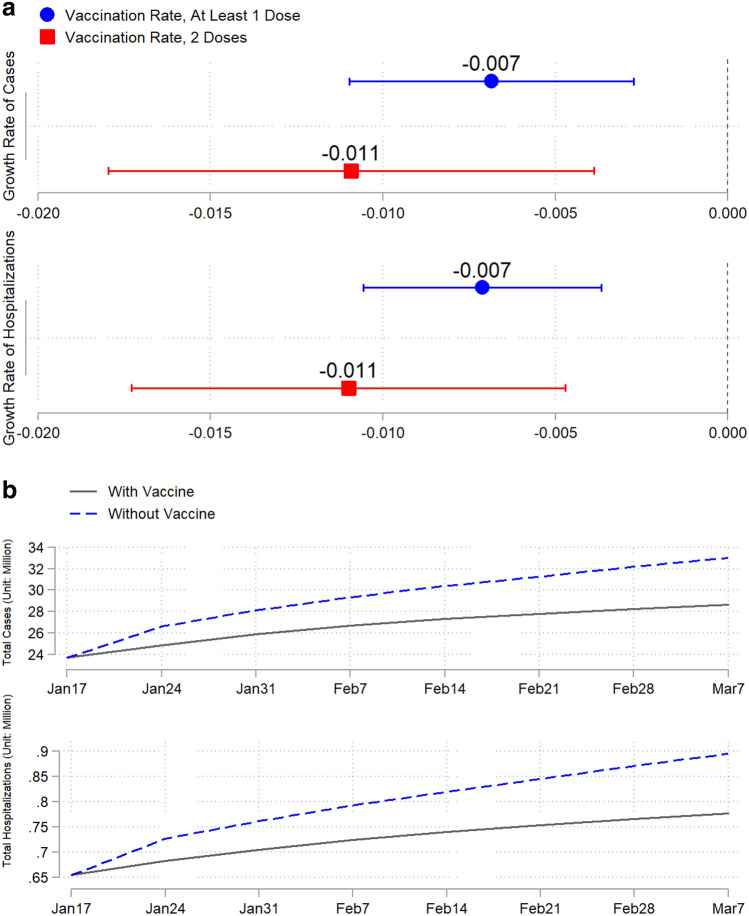
Estimated effects of vaccination on the COVID-19 pandemic. Blue markers are the estimated effects of at least 1 dose of vaccine, and red markers are the estimated effects of 2 doses of vaccine. (**a**) Baseline effect of vaccination on the growth rates of total cases and hospitalizations. (**b**) Estimated trajectories of total cases and hospitalizations without vaccines (dashed curves) versus actual trajectories of total cases and hospitalizations with vaccines (solid curves).

Vaccination has significantly slowed the growth of total COVID-19 cases and hospitalizations in the U.S. Our baseline results (Fig. 3a and Extended Data Table 2) show that one additional vaccinated individual per 100 people (at least 1 dose) reduced the growth rate of total cases by 0.7% (s.e.m. = 0.2%) and the growth rate of total hospitalizations by 0.7% (s.e.m. = 0.2%). The effects of receiving full vaccination with two doses appear greater, with reductions of 1.1% (s.e.m. = 0.4%) in the growth rate of total cases and 1.1% (s.e.m. = 0.3%) in total hospitalizations. Based on these estimates, vaccination reduced the number of new cases during our study period by 4.4 million (from 33.0 to 28.6 million), which translates into a decrease of 1.34 percentage points in the population infection rate (from 10.10% to 8.76%). Vaccination further reduced the number of hospitalizations by approximately 0.12 million, from 0.89 to 0.78 million (Fig. 3b and Supplementary Methods).

If systematic correlations existed between the pre-vaccination growth rates of infection and hospitalization and the rate of vaccination, our results would have been subject to selection bias. However, this was not the case. We demonstrated that the number of vaccines allocated to each state was proportional to its population size (Extended Data Fig. 1a). More importantly, we found that the pre-vaccination average growth rates of total cases and hospitalizations were not correlated with the average vaccination rate (Extended Data Fig. 2).

Our baseline results focus on the average treatment effect of vaccination. This effect may be heterogeneous across states that have different characteristics. For example, some evidence shows that the prevalence of COVID-19 differs across age groups, with older adults bearing the highest risk^25,26^. Because older adults were given priority during the rollout of vaccination, it is intuitive to ask whether this strategy made a difference. We separated the states into two groups according to their proportion of older adults (at least 65 years of age). Despite the slightly larger point estimate for the states with a share of older adults above the national median, the results do not differ significantly from those for the states below the median (Extended Data Fig. 3c). In addition to age, we conducted heterogeneity tests on political affiliation, nonpharmaceutical interventions, race, income, and vaccine brand. We found no significant heterogeneous effect of vaccination on any of these characteristics (Extended Data Fig. 3), implying that COVID-19 vaccines have similar effectiveness across these characteristics.

We conducted a range of sensitivity tests. First, instead of using weekly data, we ran regressions with daily data and obtained results of similar magnitudes (Extended Data Table 3). Second, we used alternative measures to capture the development of the pandemic, including the logarithms of new cases and hospitalizations and the changes in logarithms of total cases and hospitalizations. Again, using these measures, we found that vaccination has significantly slowed the pandemic (Extended Data Fig. 4 and Extended Data Table 4). Although the vaccination rollout began on 14 December 2020, our vaccination data begin 11 January 2021; we thus used linear extrapolation to impute the missing data. Our results with the inclusion of imputed data are very similar to the baseline results (Extended Data Fig. 5). Finally, we selected approximately the same number of weeks for the pre-treatment and post-treatment periods to balance the sample in our baseline results. To check the sensitivity of our results to the sample period, we ran our regressions with varying time windows, and our results remain remarkably stable. We obtained approximately the same coefficients for sample periods from 18 to 45 weeks (Extended Data Fig. 6).

**Figure 4:**
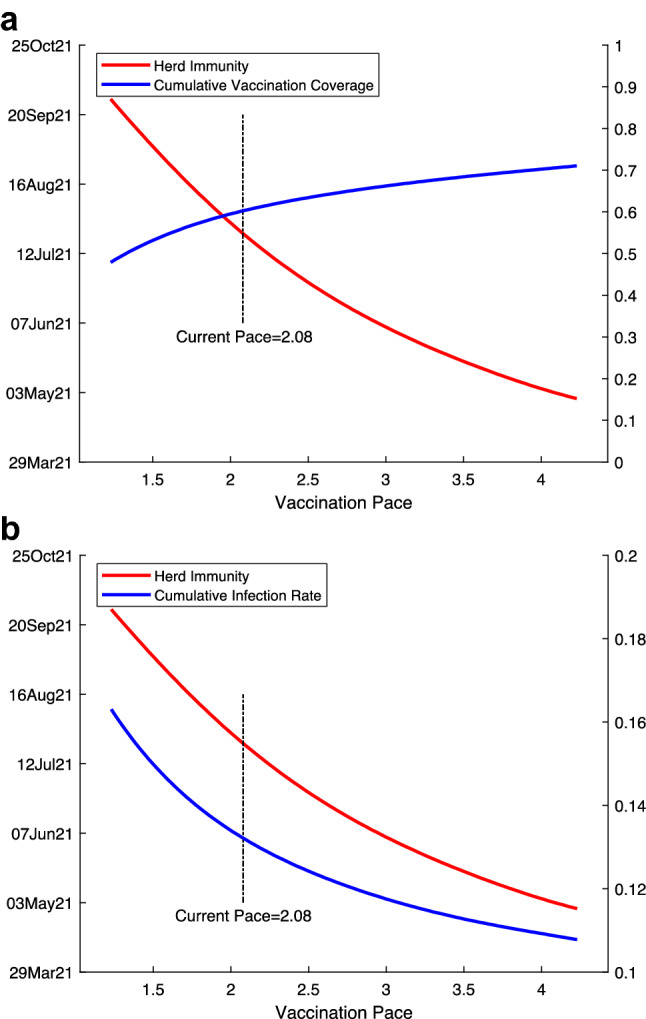
Estimated herd immunity date, cumulative vaccination coverage, and cumulative infection rate with different vaccination pace. Herd immunity date is predicted using first-dose vaccine effectiveness and first-dose vaccination pace (see "Methods"). Vaccination pace is the number of vaccine doses administered per 100 people per week. Until the first week of March 2021, the average pace over time is 2.08 doses per 100 people per week. The red curve is the predicted herd immunity date (left y-axis) in both (**a**) and (**b**). The blue curve is the estimated cumulative vaccination coverage in the population (right y-axis) when herd immunity is achieved in **a** and the estimated cumulative infection rate (right y-axis) in (**b**).

### Herd immunity

To predict how the pandemic will develop with vaccines, and especially when herd immunity might be achieved, we built a Susceptible–Infected–Recovered (SIR) model with vaccination and calibrated it to our data. We also aim to identify important factors that substantially affect the predicted date of herdy immunity. Our model predictions of the infection rate during the study period showed 99.69% correlation with the empirical data at the national level by early March 2021 (Extended Data Fig. 7). Herd immunity is achieved in the model when the real-time basic reproduction number is less than one (Supplementary Methods).

According to our model predictions, at the national average vaccination pace of 2.08 doses per 100 people per week between January and early March of 2021, the U.S. would achieve herd immunity around the last week of July 2021, with a cumulative vaccination coverage rate of 60.2% and a cumulative infection rate of 13.3%. To understand how the speed of vaccination rollout would affect the time needed to reach herd immunity, we simulated herd immunity dates by varying vaccination pace (Fig. 4). We observed a general trend that a faster vaccination pace would allow the U.S. to achieve herd immunity sooner, but with a greater number of total vaccine doses administered and a lower cumulative infection rate. This result can be explained as more individuals gaining immunity from vaccines than from infections if the vaccination pace increases.

Our predictions of herd immunity assume a continuation of vaccine uptake. In reality, however, a few potential factors could affect uptake. A certain proportion of the population might not receive the vaccination due to vaccine hesitancy. Studies have shown that vaccine hesitancy is a common phenomenon in the U.S.^27,28^, where some individuals are reluctant to receive vaccines due to the perceived risks versus benefits, certain religious beliefs, and a lack of trust in government^28^. Another issue is the effectiveness of vaccines against new coronavirus variants^29^. For example, vaccine effectiveness is lower against the Delta variant and it remains unclear how the vaccine is effective at preventing the Omicron variant^30,31^.

To examine how vaccine hesitancy and changes in vaccine effectiveness could affect our predictions for herd immunity, we incorporated in our model a range of potential vaccine hesitancy and vaccine effectiveness estimates. We assumed that if x% of the population is hesitant, then cumulative vaccination coverage in each state will stop when (1 − x%) of the population is vaccinated. Table 1 shows that a higher percentage of vaccine-hesitant individuals will lead to lower vaccination coverage with more individuals infected with COVID-19 at herd immunity. We also tested a range of vaccine effectiveness values and presented the results in Table 1. In general, higher vaccine hesitancy and lower vaccine effectiveness postpone the model-predicted herd immunity date. We further discuss the potential impact of the Delta variant on herd immunity date in the Discussion with updated data.

**Table 1:** Predicted herd immunity with different vaccination pace, vaccine hesitancy, and vaccine effectiveness estimates.

	Herd Immunity Date^a^	Vaccination Coverage^b^	Fraction Infected^b^
**Pace = 1.5**			
*Vaccine Hesitancy*^*c*^			
10%	06 Sep 2021	53.6%	15.2%
30%	06 Sep 2021	53.3%	15.2%
50%	06 Sep 2021	48.4%	16.3%
*Vaccine Effectiveness*^*d*^			
60%	27 Sep 2021	58.1%	16.7%
80%	23 Aug 2021	50.6%	14.6%
100%	26 Jul 2021	44.6%	13.3%
**Pace = 2.08 (national average pace between January and early March in 2021)**			
*Vaccine Hesitancy*			
10%	26 Jul 2021	61.4%	13.4%
30%	26 Jul 2021	60.9%	13.9%
50%	30 Aug 2021	50.0%	14.9%
*Vaccine Effectiveness*			
60%	16 Aug 2021	67.6%	14.7%
80%	12 Jul 2021	57.2%	12.9%
100%	14 Jun 2021	48.9%	11.8%
**Pace = 2.5**			
*Vaccine Hesitancy*			
10%	28 Jun 2021	63.6%	12.6%
30%	28 Jun 2021	63.1%	13.2%
50%	20 Sep 2021	50.0%	14.3%
*Vaccine Effectiveness*			
60%	26 Jul 2021	73.5%	13.8%
80%	14 Jun 2021	58.6%	12.1%
100%	24 May 2021	51.1%	11.1%

^a^Estimated week when herd immunity is achieved. The date mentioned in each row marks the first day of the week.

^b^Cumulative values when herd immunity is achieved.

^c^Percentage of the population who are hesitant to get the vaccine.

^d^Population-level effectiveness of the first dose of COVID vaccines.

## Discussion

To examine whether our main study results hold in later stages of the vaccination program, we extended our empirical analysis to 14 November 2021. Due to data limitations, we can only update one outcome measure, the growth of COVID-19 cases, but not hospitalization. As shown by Extended Data Fig. 8, vaccination (both at least one dose and two doses) is always negatively associated with the growth of total cases, but the magnitude of the estimated effect declines and the statistical significance gradually disappears with extended study time. This finding is not unexpected. First, there is evidence that the protection offered by vaccines against COVID decreases over time^19,32^. Second, the Delta variant became the dominant strain in the U.S. by mid-summer 2021. Studies have shown that the vaccines have lower effectiveness against the Delta variant^30,31^.

We also incorporated updated data on vaccine hesitancy and vaccine effectiveness in our SIR model to predict herd immunity. The weighted first-dose vaccine effectiveness is reduced to 52.28% against the Delta variant^30,31^. Based on vaccination coverage data in November, around 70% of the U.S. population received at least one dose. We thus approximated vaccine hesitancy in the population to be 30%. Additionally, researchers estimated the infectiousness of the Delta variant to be 40–60% higher than previous variants^33^, along with longer median duration (18 vs 13 days) and lower recovery rate (calculated as 1/duration)^34^. With these updated parameter estimates, our SIR model predicts the new herd immunity date to be around May 2022 (140% infection rate and 70% removal rate). We tested a range of possible values of the infection rate and removal rate (recovery rate + death rate) and show our results in Extended Data Table 4. In general, a lower removal rate tends to delay the herd immunity date, but the effect of a higher infection rate is ambiguous as agents can also get immunity via faster infection.

Our model has a few limitations. First, due to the lack of valid COVID recovery data from a few states, we imputed the missing removal rate in those states to be the national median. To address the problem of missing values, we conducted robustness checks on the removal rate. The results indicated that the national median value, which is our baseline, fits the data better, and the herd immunity date is not very sensitive to variations in removal rate (Extended Data Fig. 9). Second, our model does not consider some recent developments of the pandemic, given that it is designed to model the early stage of the vaccination program. It does not take into account new variants such as the Delta or the Omicron. Our SIR model also assumes that only susceptible individuals undergo vaccination. However, in real life, many individuals who recovered from COVID later received vaccines. The biggest limitation is the inherent assumption of an SIR model, permanent immunity, which is not true in the long run due to decreased COVID vaccine protection and the appearance of new variants. Modifying our model to an SIRS model may better capture the temporary immunity brought by COVID vaccines. That being said, our model provides valid predictions based on early-stage vaccination trends.

Some additional limitations include the discontinuation of non-pharmaceutical interventions and changes in individual attitudes/behaviors towards the pandemic. Our model assumes a continuation of the non-pharmaceutical interventions in place in early March. Relaxation of these policies would likely increase the time needed to reach herd immunity. Another issue is moral hazard, that is, whether vaccinated individuals will change their behaviors and undertake more social interaction^35^. This change could result in higher risks of infection and a delay in reaching herd immunity.

Our study provides strong evidence that vaccination has significantly decreased COVID-19 cases and hospitalizations in the U.S. Following the vaccination trends between January and early March in 2021, our model predicts that herd immunity can be achieved earlier with faster vaccination pace and lower vaccination hesitancy. However, a few factors, such as moral hazard and variants of the SARS-CoV-2 virus, could lead to changes and cast doubt as to whether herd immunity can be achieved after all.

## Methods

### Data collection and processing

A summary is provided of the data used in our analysis. Our supplementary notes give further details, including a summary statistics table for all variables.

#### Epidemiological and vaccination data

We collected our state-level epidemiological data (total COVID-19 cases, hospitalization, and tests) from the COVID Tracking Project^36^, a commonly cited source^37–39^. The vaccination data across states were obtained from the U.S. Centers for Disease Control and Prevention’s (CDC) COVID data tracker^40^, where “people vaccinated” reflects the total number of people who have received at least one vaccine dose, and “people fully vaccinated” reflects the number who have received both doses prescribed by the vaccination protocol. We downloaded the CDC vaccination data from an open-source GitHub project by Our World in Data^41^. Both the BNT162b2 (Pfizer/BioNTech) vaccine and the mRNA-1273 (Moderna) vaccine require two doses^9^. In addition, the CDC shares data on COVID-19 vaccine distribution allocations by state for both the Pfizer^42^ and Moderna^43^ vaccines, as provided by the Office of the Assistant Secretary for Public Affairs under the U.S. Department of Health & Human Services.

#### Nonpharmaceutical interventions

In addition to epidemiological data, we obtained information on nonpharmaceutical intervention policies. We adopted the policy stringency index constructed by the Oxford COVID-19 Government Response Tracker^44^, which systematically collects information on various policy responses implemented by various governments in response to the pandemic. We focused on the policy category of “containment and closure,” which covers eight policies: school closings, workplace closings, cancelation of public events, restrictions on gathering sizes, cessation of public transportation, stay-at-home requirements, restrictions on internal movement, and restrictions on international travel. This stringency index is a weighted score across these eight containment and closure policies and is scaled between 0 and 100. A detailed explanation of these measures was given by Hale et al. (2021)^45^. We determined the stringency index for each state on a weekly basis by averaging the daily data.

#### Meteorological data

Another set of important independent variables included in this study regarded the local climate. We obtained station-level hourly weather data provided by the National Centers for Environmental Information^46^. These station-level weather data were then matched with the station location and corresponding state provided by the Global Historical Climatology Network Daily^47^. We calculated the average values from these weather reports for each week across all stations within each state. Given the lack of humidity data, temperature and snow depth were used as our climate measures.

#### Election rallies and black lives matter (BLM) demonstrations

Several large-scale mass gatherings for political campaigns and protests also occurred during our study period. We constructed binary measures for election rallies^48^. For states with a rally during week *t*, this binary measure takes the value of 1 for week *t* and for the week after (*t* + 1). Our BLM data from Elephrame offered detailed information (date, location, etc.) about each demonstration from news reports^49^, which were extracted using a Web scraper. We then calculated the total number of demonstrations that occurred across all cities within each state for each week.

#### Sociodemographic data

We also collected the sociodemographic characteristics of each state’s population using 2019 estimates from the U.S. Census Bureau^50,51^. Specifically, we downloaded data on age, race, and income. We constructed each of our sociodemographic variables to be binary, above or below the national median. We derived the proportion of individuals 65 years of age and older in the population, the proportion of the white population, and the income for each state to calculate a national median. Finally, our data for the 2020 Electoral College results were obtained from the National Archives^52^. We classified the states into those won by Joe Biden and those by won by Donald Trump.

### Econometric analysis

#### Reduced-form analysis

The following reduced-form empirical model was used to estimate the impact of vaccination on the pandemic:

1 $$y_{i,t} = a_{0} + a_{1} Vaccination_{i,t - 1} + a_{2} \frac{{S_{i,t - 1} }}{{L_{i} }} + a_{3} \frac{{Test_{i,t - 1} }}{{L_{i} }} + a_{4} X_{i,t - 1} + b_{i} + c_{t} + \varepsilon_{i,t} .$$

Here, ${y}_{it}$ is the dependent variable that measures the growth of either total cases or total hospitalizations in state *i* at period *t*. Our baseline measure is the growth rate, which is defined as $\frac{{{C}_{i,t}-C}_{i,t-1}}{{C}_{i,t-1}}$ for total cases and $\frac{{{H}_{i,t}-H}_{i,t-1}}{{H}_{i,t-1}}$ for total hospitalizations, where ${C}_{i,t}$ and ${H}_{i,t}$ are the cumulative numbers of cases and hospitalizations. Alternative outcome measures were also used in the sensitivity analysis (Extended Data Fig. 4).

Our key independent variable, ${Vaccination}_{i,t-1}$, is the rate of vaccination of state *i* in period *t-*1, and ${a}_{1}$ is the coefficient of interest. We used two measures of vaccination rate: the number of vaccinated people (i.e., those who had received at least one dose of vaccine) per hundred and the number of fully vaccinated people (i.e., those who had received two doses of vaccine) per hundred. As the proportion of susceptible individuals in the total population decreases over time, the growth rate of infection may also decline. To deal with this intrinsic dynamic, ${S}_{i,t-1}/{L}_{i}$ was included in the regression model to control for the stock of susceptible individuals ${S}_{i,t-1}$ in the total population ${L}_{i}.$ We measured ${S}_{i,t-1}$ as the difference between the population size and the total number of infections. To adjust for differences in testing intensity across states, we added ${Test}_{i,t-1}/{L}_{i}$ to control for the number of tests relative to the total population.

Our control variables, ${X}_{i,t}$, contain a dummy variable ${rally}_{i,t}$, which equals 1 when an election rally occurred in state *i* at period *t*. We also added a variable ${protest}_{i,t}$, which is the number of protests held across all cities in state *i* at period *t*. To capture the influence of climate on the pandemic, we included measures of state-level meteorological conditions, including average temperature, temperature deviation from the state mean, and the logarithm of the average snow depth. Note that we included state fixed effects (${b}_{i}$) to capture state-specific unobserved factors, which are time-invariant, such as location, geography, and culture. We also included week fixed effects $\left({c}_{t}\right)$ to capture unobserved shocks, which are common across states, such as macroeconomic conditions. Finally, ${\varepsilon }_{i,t}$ is a random error term of the model, which has a mean of zero.

We estimated Eq. (1) using the method of Ordinary Least Square with weekly data for 50 states and DC in the baseline. Robust standard errors for the estimated coefficients with two-way clustering were calculated at the state and week levels^53^. Therefore, we allowed for within-state autocorrelation in the error term to capture the persistence of the pandemic within each state. We also allowed for spatial autocorrelation in the error term to capture common pandemic shocks or systematic misreporting across states.

#### Model summary

We modified a conventional SIR model with the addition of vaccination to simulate the development of the COVID-19 pandemic in the U.S. with vaccine rollout, including both state-level and national-level estimates. The theoretical SIR model with vaccination is as follows:

2 $$\begin{gathered} \frac{{dS_{i,t} }}{dt} = - \beta_{i,t} S_{i,t} I_{i,t} - e\delta_{i,t} , \hfill \\ \frac{{dI_{i,t} }}{dt} = \left( {\beta_{i,t} S_{i,t} - \gamma_{i} } \right)I_{i,t} , \hfill \\ \frac{{dR_{i,t} }}{dt} = \gamma_{i} I_{i,t} + e_{t} \delta_{i,t} . \hfill \\ \end{gathered}$$

Here, ${S}_{i,t}$ is the state-specific (*i*) and time-varying (*t*) proportion of susceptible individuals in the population, ${I}_{i,t}$ is the proportion of infected individuals, and ${R}_{i,t}$ the proportion of recovered (plus dead) individuals. ${\beta }_{i,t}$ is the infection rate, which determines the spread of the pandemic. ${\gamma }_{i}$ includes both recovered individuals and deaths and is referred to as the removal rate^5^. Here ${\gamma }_{i}$ varies only by state and not over time. ${\delta }_{i,t}$ is the proportion of vaccinated individuals, and ${e}_{t}$ is the population-level vaccine effectiveness, which remains the same across states but may change in simulations to capture the effect of new variants.

We fit the SIR model above with state-level COVID-19 epidemiology data, from which we observed data on the cumulative number of cases, deaths, and vaccination doses administered. Only 29 of the 51 states (counting DC as a “state” for this purpose) reported valid recovery data. We imputed the missing data for the other 22 states with the median recovery and mortality rates from the known 29 states (see Supplementary Methods for details). We first estimated the infection rate (${\beta }_{i,t}$) and vaccination coverage (${\delta }_{i,t}$). To capture the impact of nonpharmaceutical interventions on the spread of COVID-19^4–6^, we used the following equation to estimate the infection rate with state fixed effect (${\rho }_{i}$) and time fixed effect ($\rho_{t}$):

3 $${\upbeta }_{{{\text{i}},{\text{t}}}} = \theta_{0} + \theta_{1} \cdot policy_{i,t} + \rho_{i} + \rho_{t} + \varepsilon_{i,t}^{\beta }$$

Similarly, we estimated vaccination coverage using the following equations, controlling for state and time fixed effects.

4 $${\updelta }_{{{\text{i}},{\text{t}}}} = \eta_{0} + \iota_{i} + \iota_{t} + \varepsilon_{i,t}^{\delta }$$

We adopted two vaccination measures in our data: the total number of people who had received at least one vaccine dose and the total number of fully vaccinated people. No time trends were observed in the total doses administered for at least one dose of vaccine, but an apparent time trend was seen in the doses administered for the second dose. We therefore added a time trend in the estimation equation above when we conducted the sensitivity check using the total number of fully vaccinated people as our measure of vaccination. We used Eqs. (3) and (4) to estimate the infection rate and vaccination coverage, combined with the initial epidemiological data of SIR in week 1 (12 October 2020), and our model estimates of the infection rate for the following 20 weeks are highly correlated with the empirical data. For each individual state, our model estimates reached a median correlation of 99.04% (range, 86.37% to 99.95%) (Extended Data Fig. 7).

We assessed herd immunity based on our model estimates of the real-time basic reproduction number for each state, ${R{^{\prime}}}_{i,t}=\frac{{\upbeta }_{\mathrm{i},\mathrm{t}}{S}_{i,t}}{{\gamma }_{i}}$; that is, the number of cases directly caused by an infected individual throughout his or her infectious period. The model achieves herd immunity when ${R{^{\prime}}}_{i,t}$ falls below 1 in 49 states (except for Maryland and Kentucky; see Supplementary Methods for details).

For each given vaccination pace, we ran the simulation forward and projected the future dynamic of the pandemic across the U.S., assuming that no changes are made in nonpharmaceutical interventions. We then computed the time required for every state to achieve herd immunity and calculated the share of the U.S. population vaccinated when herd immunity is achieved. In addition, we conducted a sensitivity analysis regarding herd immunity with variations in vaccine effectiveness and with the addition of vaccine hesitancy. We incorporated vaccine hesitancy into our model by assuming that if x% of the population is hesitant, the cumulative vaccination coverage in each state will stop when (1 − x%) of the population is vaccinated.

## Supplementary Information

Supplementary Information 1.

Supplementary Information 2.

## Data Availability

The datasets and code used for the analyses are available at https://github.com/huntabaobao007/US-COVID-19-vaccination.
