# Supplementary material for: Impact of vaccination on the COVID-19 pandemic in U.S. states
Source: Sci Rep. 2022 Jan 28;12:1554. doi: 10.1038/s41598-022-05498-z (PMC8799714; doi:10.1038/s41598-022-05498-z)
Supplement: Supplementary file 1 — Supplementary Information 1. [file 41598_2022_5498_MOESM1_ESM.docx]

**Supplementary Information**

The supplementary information provides supplementary methods, figures, and tables.

**1. Supplementary Methods**

**1.1 Estimating the impact of vaccination**

We used our reduced-form estimates to carry out back-of-the-envelope calculations to derive the number of new cases prevented by vaccination. For this purpose, we first calculated the counterfactual growth rate of total cases by $\hat{y}_{i,t}={y_{i,t}-\hat{a}_{1}Vaccine}_{i,t-1}$ with $\hat{a}_{1}$ and ${Vaccine}_{i,t-1}$. $\hat{a}_{1}$ is the estimated effect of vaccination on the growth rate of total cases, and ${Vaccine}_{i,t-1}$ is the observed vaccination rate for the previous period. We know that the growth rate $y_{it}$ satisfies $C_{i,t}=C_{i,t-1}(1+y_{i,t})$, where $C_{i,t}$ is the observed number of total cases. With the series of counterfactual growth rates of total cases {$\hat{y}_{i,t}{\}}_{t=1}^{T}$ (*T* is the last period of the sample) and a given initial value of total cases ($C_{i,0}$) before vaccination began, we can infer a counterfactual (without vaccination) series of total cases {$\hat{C}_{i,t}{\}}_{t=1}^{T}$ using

$\hat{C}_{i,t-1}=C_{i,0}$,

$$\hat{C}_{i,t}=\hat{C}_{i,t-1}\left( 1+\hat{y}_{i,t} \right).$$

Summing across 50 states and DC, the impact of vaccination on the number of total cases ($\Delta C$) is given by

$\Delta C=\sum_{i=1}^{51} \left( \hat{C}_{i,T}-C_{i,T} \right)$.

Repeating this process with hospitalization data, we evaluated the impact of vaccination on the total number of hospitalizations during our sample period.

Our extended analysis further examining the vaccinnaton effectiveness used updated data till 14 Nov 2021. However, there are two data limitations. First, we can only examine the growth of total cases but not total hospitalizations. Second, we cannot control for testing. Our reduced-form empirical model now takes the following form:

| $y_{i,t}=a_{0}+a_{1}{Vaccination}_{i,t-1}+a_{2}X_{i,t-1} +b_{i}+c_{t}+\varepsilon_{i,t}$ |
| --- |

where $y_{it}$ is the dependent variable that measures the growth of total cases in state *i* at period *t*, ${Vaccination}_{i,t-1}$, is the rate of vaccination of state *i* in period *t-*1. We used two measures of vaccination rate: the number of vaccinated people per hundred and the number of fully vaccinated people per hundred. $X_{i,t}$, continues to include as controls such as public gatherings and meteorological conditions, but not testings, as mentioned above.

We estimated the above equation via OLS with weekly data for 50 states and DC. Similar to our baseline in the manuscript, we set the start date at 12 October 2020 and gradually expanded the time window week by week from 7 March to 2 May 2021, and then every four weeks afterward from 30 May to 14 November.

**1.2 Estimation of Model Parameters**

Here we provide more details on parameter estimation for our SIR model with vaccination. We used state-level weekly epidemiological and vaccination data for the estimation during the period from 12 October 2020 to 7 March 2021. The data demonstrate the cumulative population share of infected individuals $I_{i,t}^{cum}$ and COVID-19–related deaths $D_{i,t}^{cum}$ for all 50 states and DC, and valid recovery data $R_{i,t}^{cum}$ for 29 states (The states with valid recovery data are AL, AR, DC, ID, KY, LA, ME, MD, MA, MI, MN, MS, MT, NE, NH, NM, ND, OH, OK, PA, SC, SD, TN, TX, UT, VT, WV, WI, and WY. Although IA also reported recovery, the number was higher than the cumulative number of infections. We therefore excluded IA as well). For states with recovery data, we calculated the proportion of infected, susceptible, recovered, and dead individuals for the current period *t* using the following equations: $I_{i,t}^{cur}= I_{i,t}^{cum}- R_{i,t}^{cum}- D_{i,t}^{cum}$, $S_{i,t}^{cur}=1- I_{i,t}^{cum}- {eV}_{i,t}^{cum}$, $R_{i,t}^{cur}=R_{i,t}^{cum}-R_{i,t-1}^{cum}$ and $D_{i,t}^{cur}=D_{i,t}^{cum}-D_{i,t-1}^{cum}$, respectively.

**Removal rate ($\boldsymbol{\gamma}_{\mathbf{i}}$)** In equation (2), $\gamma_{i}$ stands for the removal rate from the infection group. We calculated a state-specific but time-invariant $\gamma_{i}$ by considering both recovered individuals and deaths following Hsiang et al. (2020). We obtained complete death data over the study period, but valid recovery data are available for only 29 states. Therefore, we first calculated the average recovery and mortality rates in the 29 states for which we have valid recovery data as

$$\gamma_{i}=\frac{1}{T}\sum_{t=1}^{T} \frac{D_{i,t}^{cur}+R_{i,t}^{cur}}{I_{i,t}^{cur}},$$

where *T* is the number of weeks in the sample period. The removal rates in the remaining 22 states were assumed to be the median of the removal rates in the 29 states for which complete recovery data are available, that is, 30.15% (we used the median instead of the mean to dampen the influence of outliers, similar to Hsiang et al, 2020). To check the robustness, we imputed missing values with 25^th^ and 75^th^ percentile estimation rather than median, the result showed that 25^th^ percentile removal rate will overestimate cumulative infection while 75^th^ percentile one will underestimate, both in considerable extent. But the prediction on herd immunity date rarely changed (Extended Data Fig. 9).

**Infection rate ($\boldsymbol{\beta}_{\mathbf{i,t}}$)** In our SIR model, $\beta_{i,t}$ determines the spread of the pandemic. According to equation (2), we have

$\beta_{i,t}=max\{\frac{I_{i,t+1}^{cur}/I_{i,t}^{cur}-1+\gamma_{i}}{S_{i,t}^{cur}},0\}$,

which we used to calculate $\beta_{i,t}$ in the 29 states that we have recovery data to derive the removal rate $\gamma_{i}$ directly. To estimate $\beta_{i,t}$ for the other 22 states with no recovery data, we first assumed that $\beta_{i,t}$ is determined by the stringency of nonpharmaceutical interventions and used the following reduced-form equation,

$\beta_{i,t}=\theta_{0}+\theta_{1}\cdot{policy}_{i,t}+\rho_{i}+\rho_{t}+\varepsilon_{i,t}^{\beta}$,

which estimates $\beta_{i,t}$ for the 29 states with recovery data using the observed nonpharmaceutical interventions, along with state fixed effects ($\rho_{i}$) and time fixed effects ($\rho_{t}$). We then inferred $\beta_{i,t}$ for the remaining 22 states based on the estimated $\theta_{1}$, the observed policies, and the median estimates of state and time fixed effects. We also assumed that future non-pharmaceutical interventions would remain at the same level as in the last week of our sample (i.e., the week of March 1, 2021) when generating model predictions.

**Vaccination rate ($\boldsymbol{\delta}_{\mathbf{i,t}}$)** We calculated the population share of newly vaccinated people by $\delta_{i,t}={(V}_{i,t}^{cum}-V_{i,t-1}^{cum})/L_{i}$, where $L_{i}$ is the total population size in state . We then estimated $\delta_{i,t}$ with state fixed effects and time fixed effects. Specifically, we used $\delta_{i,t}^{(1)}=\eta_{0}^{(1)}+\iota_{i}^{(1)}+\iota_{t}^{(1)}+\varepsilon_{i,t}^{\delta^{(1)}}$ to estimate the vaccination rate for the first dose and $\delta_{i,t}^{(2)}=\eta_{0}^{(2)}+\eta_{1}^{(2)}t+\iota_{i}^{(2)}+\iota_{t}^{(2)}+\varepsilon_{i,t}^{\delta^{(2)}}$ for the second dose. We predicted $\delta_{i,t}$ for each state in future periods based on the estimated constants ($\eta_{0}^{(1)}$, $\eta_{0}^{(2)}$), coefficient ($\eta_{1}^{(2)}$), state fixed effects ($\iota_{i}^{(1)}$, $\iota_{i}^{(2)}$), and the median of time fixed effects ($\iota_{t}^{(1)}$, $\iota_{t}^{(2)}$).

**Vaccine efficacy (e)** According to previous studies focusing on the alpha variant, the Pfizer vaccine has an efficacy of 52.0% after the first dose, and the Moderna vaccine has an efficacy of 92.1% after the first dose (Creech et al, 2021). Pfizer occupied a share of 47.75% of U.S. vaccine distribution during our sample period, and Moderna occupied the rest. We thus calculated the overall efficacy before 7 March 2021 as the weighted average of both vaccines, at 73.0%. Extended analysis with the Delta variant adopted a first-dose efficacy of 30.7% for Pfizer and 72% for Moderna. Hence we derived the overall efficacy at 52.28% after 7 March 2021.

**Missing data imputation** Due to the lack of recovery data, we only know the cumulative infection rate rather than the current infection rate in the 22 states for which recovery data are missing. To produce quantitative results as accurately as possible, we used our SIR model to impute missing data for these 22 states. We first estimated vaccination coverage $\hat{\delta}_{i,t}$ and the infection rate $\hat{\beta}_{i,t}$ for these 22 states. Then, given the current infection rate at the initial period $\hat{I}_{i,1}^{cur}$, we calculated $\hat{S}_{i,1}^{cur}$ and generated the dynamics of cumulative infection rate $\{\hat{I}_{i,t}^{cum}\}$ using

$\hat{I}_{i,t+1}^{cur}=\hat{I}_{i,t}^{cur}(1-\hat{\gamma}_{i}+\hat{\beta}_{i,t}\hat{S}_{i,t}^{cur})$,

$$\hat{S}_{i,t+1}^{cur}=\hat{S}_{i,t}^{cur}\left( 1-\hat{\beta}_{i,t}\hat{I}_{i,t}^{cur} \right)-e\hat{\delta}_{i,t},$$

$\hat{I}_{i,t+1}^{cum}=\hat{I}_{i,t}^{cum}+\hat{\beta}_{i,t}\hat{S}_{i,t}^{cur}\hat{I}_{i,t}^{cur}$.

We then matched the model generated $\{\hat{I}_{i,t}^{cum}\}$ from the equations above for each of the 22 states with observed data for cumulative infection $\left\{ I_{i,t}^{cum} \right\}$ by minimizing the loss function below

$${min \atop\{I_{i,1}^{cur}\}}\sum_{t=1}^{T} \left( \frac{\hat{I}_{i,t}^{cum}}{I_{i,t}^{cum}}-1 \right)^{2}.$$

Supplementary Table 1 shows estimated region fixed effects. Supplementary Table 2 presents the estimated time fixed effects. Other model parameter input values can be found in Supplementary Table 3.

**1.3 Model Fit**

We examined how well our calibrated model fits the empirical data. The infection rates predicted by our model match the general trend in the U.S. and in most states quite well (see Supplementary Table 1 and Extended Data Fig. 7); the average correlation was 99.69% at the national level. Table 1 compares the fitness of the model results with the empirical data for each state. There were two exceptions, Kentucky (KY) and Maryland (MD), for which our model predictions were off-target by relatively large margins. However, this was due to the estimated removal rates $(\gamma_{i})$ for these two states, which are outliers (Supplementary Fig. 1).

**1.4 Model Predictions and Herd Immunity**

The basic reproduction number, $R'=\frac{\beta}{\gamma},$ is the key measure used to assess the dynamics of the pandemic and to calculate the vaccination coverage to achieve herd immunity (Sun, 2010; Sun and Shi, 2011). It is worthy of note that this formula only applies during the early stage of disease when the susceptible density approaches 1. However, at a later stage of the pandemic and with vaccines, a considerable share of the susceptible population has been vaccinated or has recovered, so the share of susceptible individuals can be significantly less than 1. According to the definition of the basic reproduction number, at period *t*, an infected person is expected to infect $\beta_{t}S_{t}$ people with an expected duration infection time of $1/\gamma$. Therefore, the time-varying reproduction number is ${R'}_{t}=\beta_{t}S_{t}/\gamma$. At the beginning of the pandemic, we have $t=0$, $S_{t}=1$ and $R_{0}=\beta_{0}/\gamma,$ which is consistent with the conventional definition. To assess whether the U.S. as a whole has acquired herd immunity, we use the “Third Statistics” approach; that is, the third-worst state’s reproduction number is used to form the national level “reproduction number”:

$${R'}_{t}={{max}_{3} \atop i=1,\ldots,51}\left\{ {R'}_{i,t} \right\}.$$

We used this measure to rule out the impact of outliers (Eaton and Kortum, 2002). As Supplementary Fig. 1 indicates, two states (Kentucky and Maryland) reported unreasonably low recovery numbers, which greatly biased our calculations of the reproduction number.

**Supplementary Fig. 1|** **Boxplot for estimated state-level recovery rate.** This figure plots the distribution of the estimated recovery rate for the 29 states with valid recovery data during our study period. According to the boxplot, Kentucky (KY) and Maryland (MD) appear as outliers.

**Supplementary Table 1. State fixed effects and model fitness across all 50 states and DC**. (in attached Excel file due to table size)

**Supplementary Table 2. Time fixed effects across all 50 states and DC**

|  | **Estimated with First-Dose Data** | | **Estimated with Second-Dose Data** | |
| --- | --- | --- | --- | --- |
| **Time** | **Infection Rate Fixed Effect** $\boldsymbol{\rho}_{\mathbf{t}}$ **(%)** | **Vaccination Rate**  **Fixed Effect** $\boldsymbol{\iota}_{\mathbf{t}}^{\boldsymbol{(1)}}$ **(%)** | **Infection Rate**  **Fixed Effect** $\boldsymbol{\rho}_{\mathbf{t}}$ **(%)** | **Vaccination Rate**  **Fixed Effect** $\boldsymbol{\iota}_{\mathbf{t}}^{\boldsymbol{(2)}}$ **(%)** |
| Week 1 | 0.00 | N/A | 0.00 | N/A |
| Week 2 | 3.83 | N/A | 3.83 | N/A |
| Week 3 | 8.91 | N/A | 8.91 | N/A |
| Week 4 | 16.35 | N/A | 16.36 | N/A |
| Week 5 | 2.41 | N/A | 2.44 | N/A |
| Week 6 | -7.23 | N/A | -7.18 | N/A |
| Week 7 | -5.49 | N/A | -5.43 | N/A |
| Week 8 | -7.32 | N/A | -7.27 | N/A |
| Week 9 | -12.08 | N/A | -12.02 | N/A |
| Week 10 | -16.36 | N/A | -16.30 | N/A |
| Week 11 | -11.68 | N/A | -11.62 | N/A |
| Week 12 | -3.07 | N/A | -3.01 | N/A |
| Week 13 | -12.70 | N/A | -12.66 | N/A |
| Week 14 | -18.39 | 0.00 | -19.14 | 0.00 |
| Week 15 | -21.14 | -1.08 | -22.20 | -0.21 |
| Week 16 | -22.29 | -1.46 | -23.50 | -0.13 |
| Week 17 | -24.19 | -1.58 | -25.41 | -0.02 |
| Week 18 | -24.66 | -1.47 | -25.95 | 0.10 |
| Week 19 | -21.73 | -0.83 | -23.30 | 0.33 |
| Week 20 | -15.15 | -1.27 | -17.04 | 0.23 |
| Week 21 | N/A | -0.63 | N/A | 0.00 |

**Supplementary Table 3. Other model parameter values.**

|  | **Notations** | **Values** |
| --- | --- | --- |
| **Estimations with**  **First Dose Data** | Constant of Infection Rate Estimation ($\theta_{0}$) | 0.391 |
|  | Elasticity of Policy on Infection Rate Estimation ($\theta_{1}$) | 0.00529 |
|  | Constant of Vaccination Rate Estimation ($\eta_{0}^{(1)}$) | 0.0285 |
| **Estimations with Second Dose Data** | Constant of Infection Rate Estimation ($\theta_{0}$) | 0.397 |
|  | Elasticity of Policy on Infection Rate Estimation ($\theta_{1}$) | -0.00542 |
|  | Constant of Vaccination Rate Estimation ($\eta_{0}^{(2)}$) | 0.00142 |
|  | Trend of Vaccination Rate Estimation ($\eta_{1}^{\left( 2 \right)}$) | 0.00185 |

**Reference**

Creech, C. Buddy, Shannon C. Walker, and Robert J. Samuels. SARS-CoV-2 vaccines. *JAMA* 325, no. 13 (2021): 1318-1320.

Eaton, Jonathan, and Samuel Kortum. Technology, geography, and trade. *Econometrica* 70.5 (2002): 1741-1779.

Hsiang, Solomon, et al. The effect of large-scale anti-contagion policies on the COVID-19 pandemic. *Nature* 584.7820 (2020): 262-267.

Sun, Ruoyan. Global stability of the endemic equilibrium of multigroup SIR models with nonlinear incidence. *Computers & Mathematics with Applications* 60.8 (2010): 2286-2291.

Sun, Ruoyan, and Junping Shi. Global stability of multigroup epidemic model with group mixing and nonlinear incidence rates. *Applied Mathematics and Computation* 218.2 (2011): 280-286.

**Acknowledgments**

We thank K.E. Warner and S. Mennemeyer for their feedback.

**Author contributions**

All authors designed the analyses, interpreted the results, and designed the figures, and are listed alphabetically. X.C., H.H., R.S., and J.Z. contribute equally to the paper. H.H. and R.S. collected the data. J.Z. conducted the reduced-form empirical analysis. X.C. conducted the analysis with the SIR model. H.H., J.J., and R.S. wrote the paper.

**Funding**

H.H. is funded by the startup grant from the City University of Hong Kong and the Research Grant Council of Hong Kong (project number 11501121). X. C. is funded by the Project 72003026 supported by NSFC and the Project 19QD01 supported by the Fundamental Research Funds for the Central Universities in UIBE.

**Competing interests**

H.H., X.C., J.J, R.S., and J.Z. declare no conflicts of interest.

**Extended Data**

a.


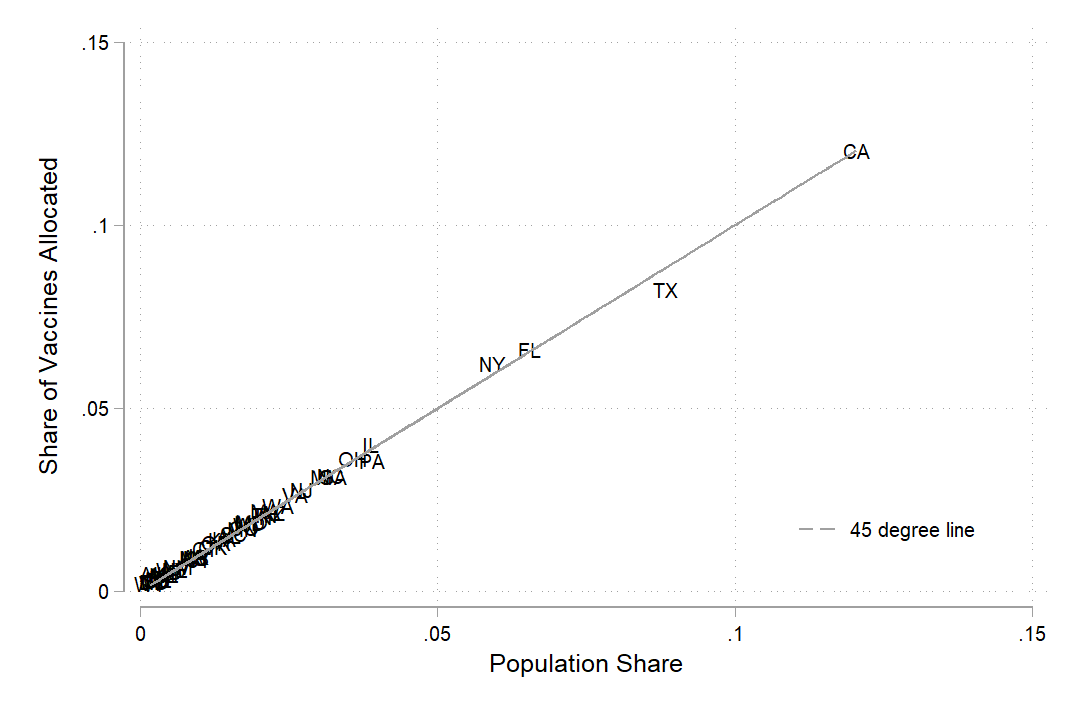


b.


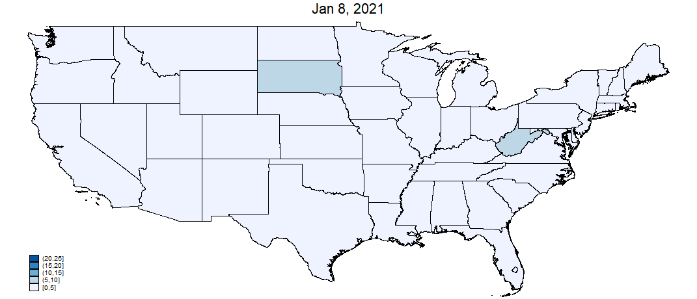

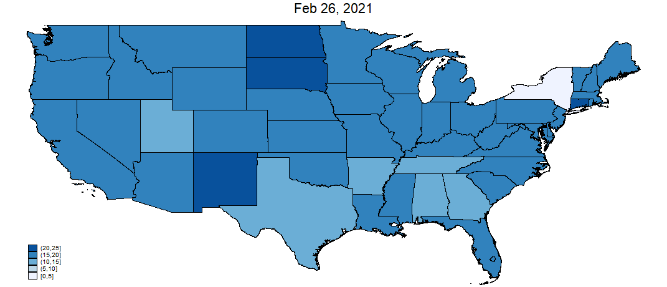


**Extended Data Fig. 1**| **COVID-19 vaccination in all 50 U.S. states and DC.** **a**, Share of vaccines allocated versus population share. **b**, Heat map of vaccines administered by states over time. The darker the color, the more doses of vaccines administered per 100 people.


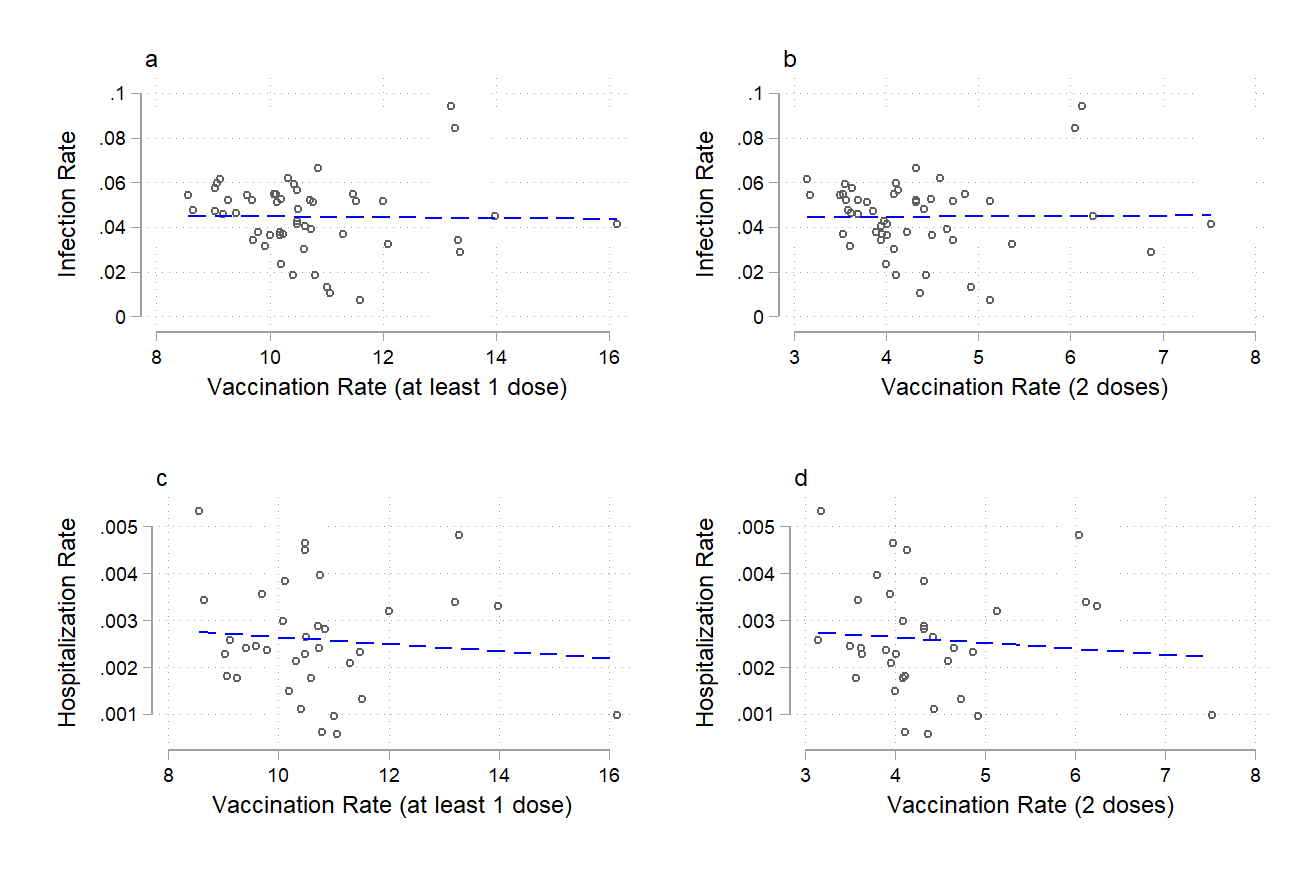


**Extended Data Fig. 2|** **COVID-19 infections (average total infection and hospitalization rates) before vaccination and average vaccination rate.**  **a,** Association between the total infection rate before vaccination and at least 1 dose of vaccination (coefficient = 0.0002, R^2^ = 0.0%). **b,** Association between the total infection rate before vaccination and 2 doses of vaccination (coefficient = 0.0002, R^2^ = 0.0%). **c,** Association between the total hospitalization rate before vaccination and at least 1 dose of vaccination (coefficient = 0.0000, R^2^ = 1.0%). **d,** Association between the total hospitalization rate before vaccination and 2 doses of vaccination (coefficient = 0.0001, R^2^ = 0.9%).


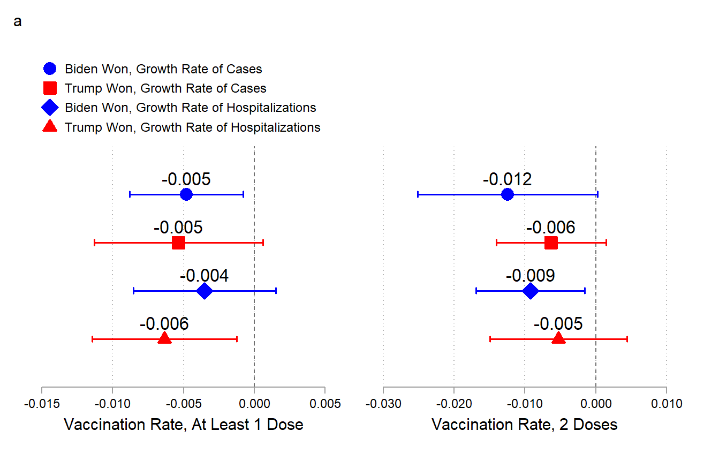

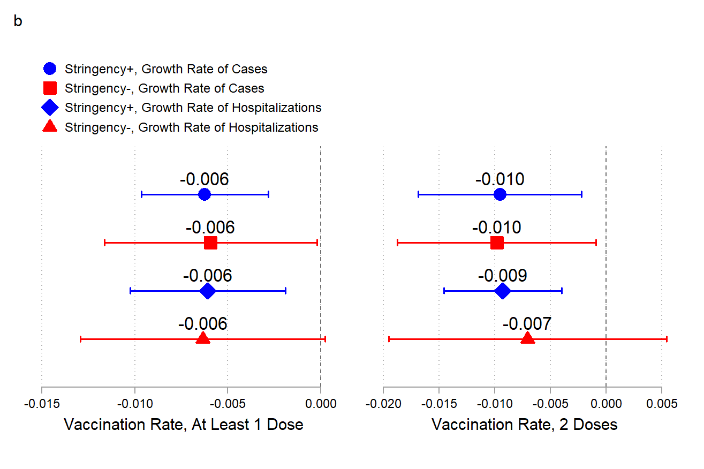

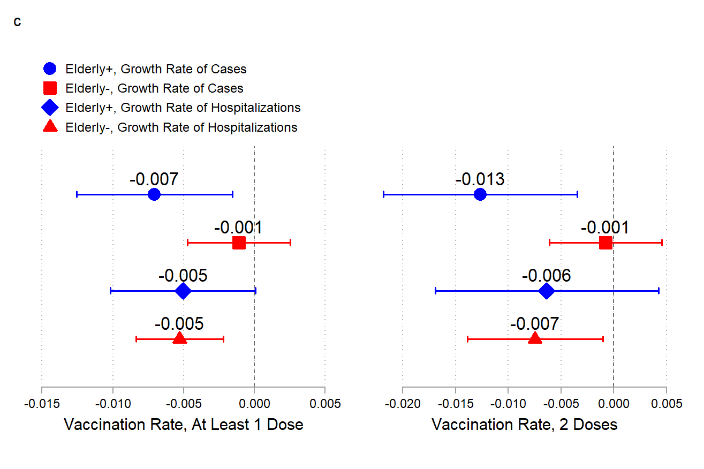

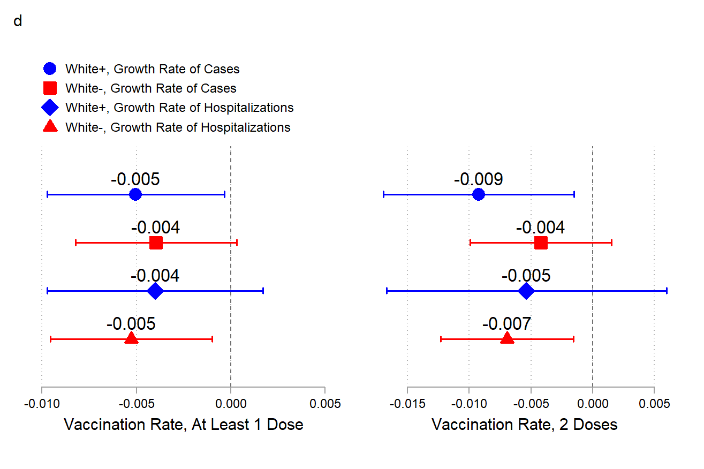

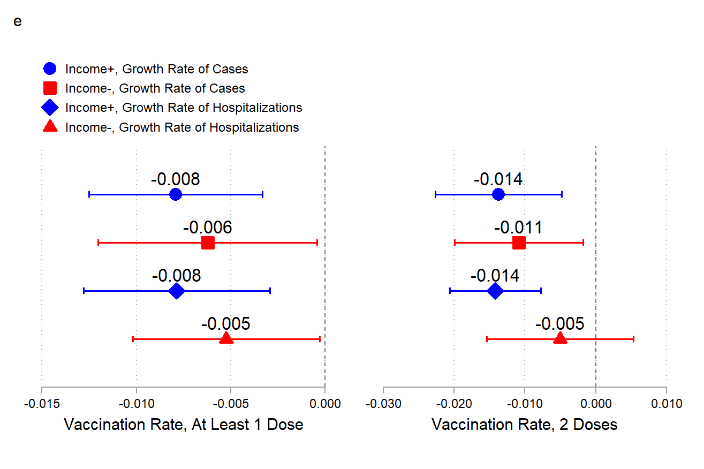

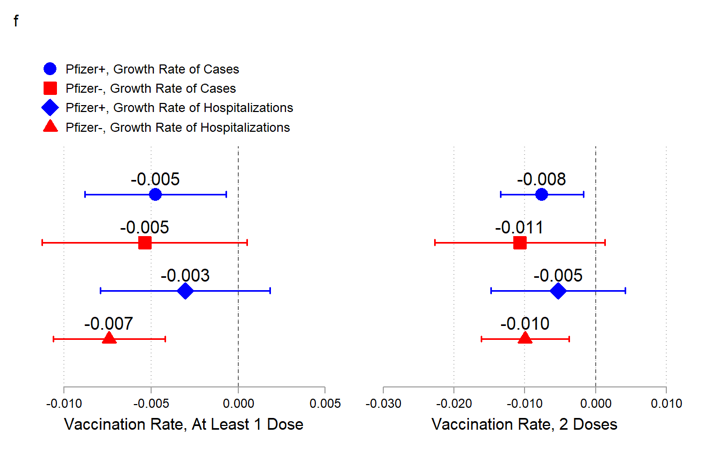


**Extended Data Fig. 3|** **Heterogeneity tests on the effect of vaccination across various state characteristics.** Blue markers are the estimated effects of at least 1 dose of vaccine, and red markers are the estimated effects of 2 doses of vaccine. **a,** Effect of vaccination in states where the 2020 presidential election was won by Joe Biden versus Donald Trump. **b,** Effect of vaccination in states with non-pharmaceutical interventions more stringent than the national median (+) versus less stringent than the median (-). **c,** Effect of vaccination in states with the proportion of the elderly population (65+) greater than the national median (+) versus less than the median (-). **d,** Effect of vaccination in states with the proportion of the white population greater than the national median (+) versus less than the median (-). **e,** Effect of vaccination in states with per capita income greater than the national median (+) versus less than the median (-). **f,** Effect of vaccination in states with the share of Pfizer vaccine greater than the national median (+) versus less than the median (-).


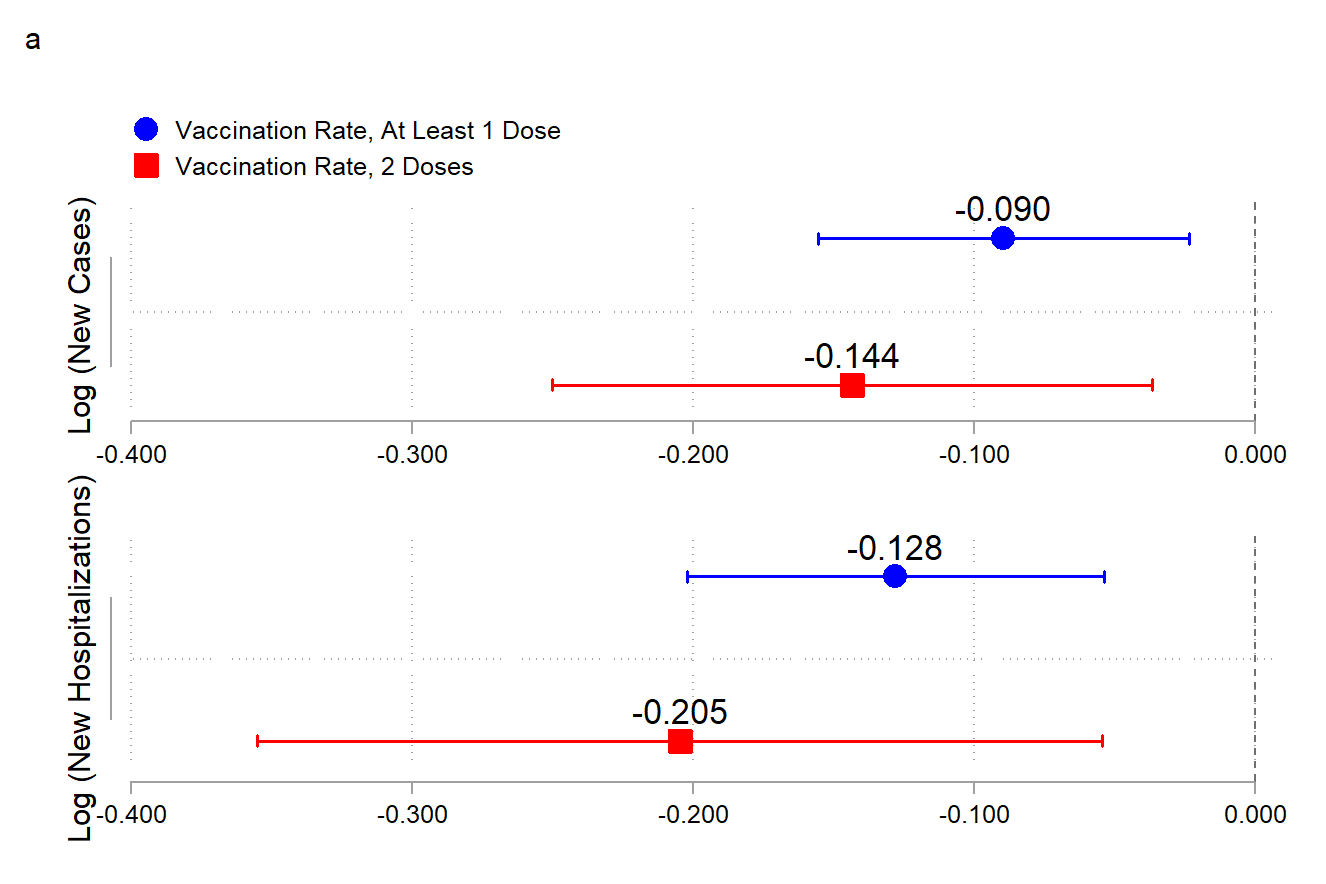


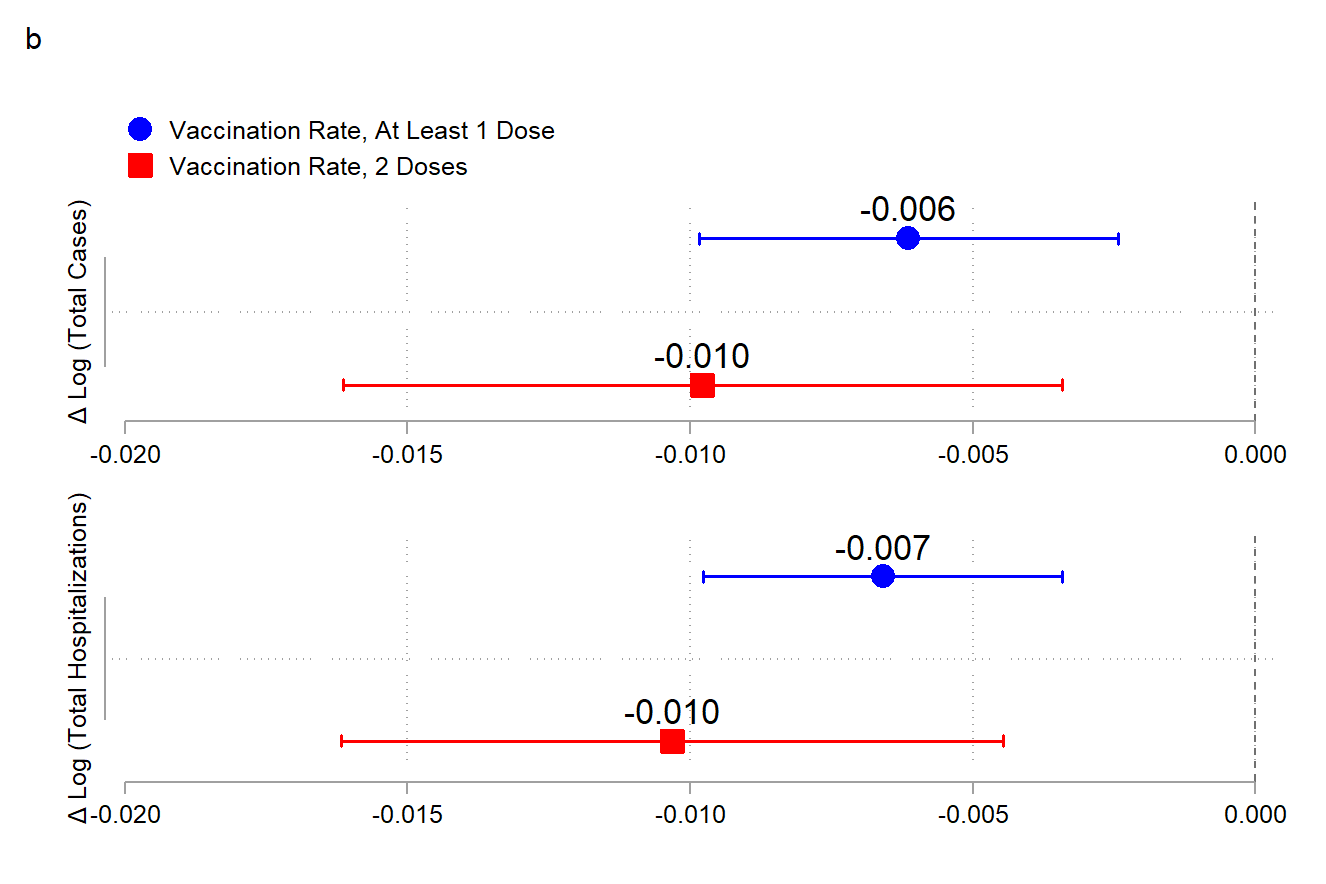


**Extended Data Fig. 4| Estimated effects of vaccination on the COVID-19 pandemic with alternative outcome measures.** Blue markers are the estimated effects of at least 1 dose of vaccine, and red markers are the estimated effects of 2 doses of vaccine. **a,** Estimated effects of vaccination on logarithms of news cases and hospitalizations. **b,** Estimated effects of vaccination on changes in logarithms of total cases and hospitalizations.


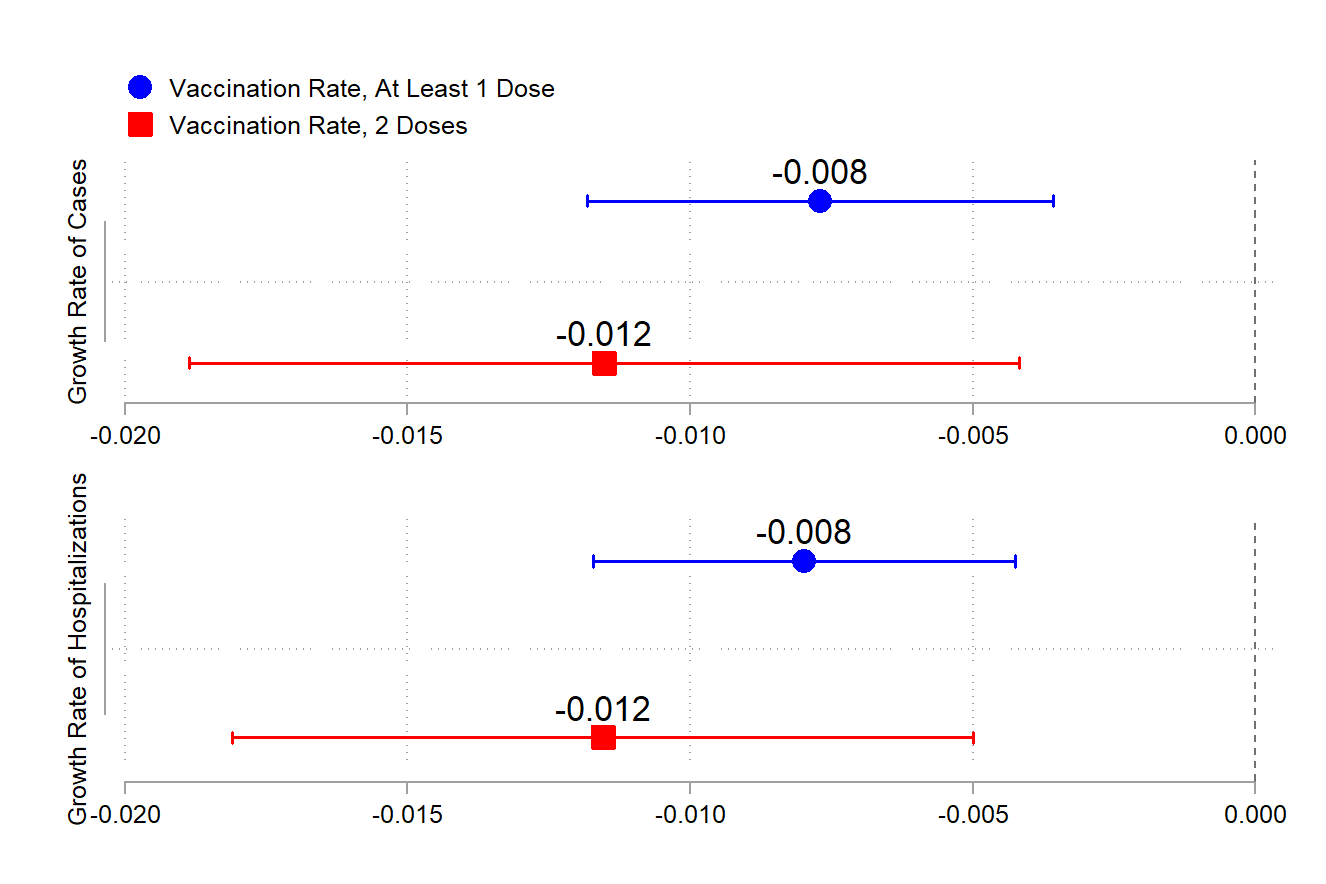


**Extended Data Fig. 5| Estimated effects of vaccination on the COVID-19 pandemic with imputed missing data on vaccination between 21 December 2020 and 10 January 2021.** Blue markers are the estimated effects of at least 1 dose of vaccine, and red markers are the estimated effects of 2 doses of vaccine.


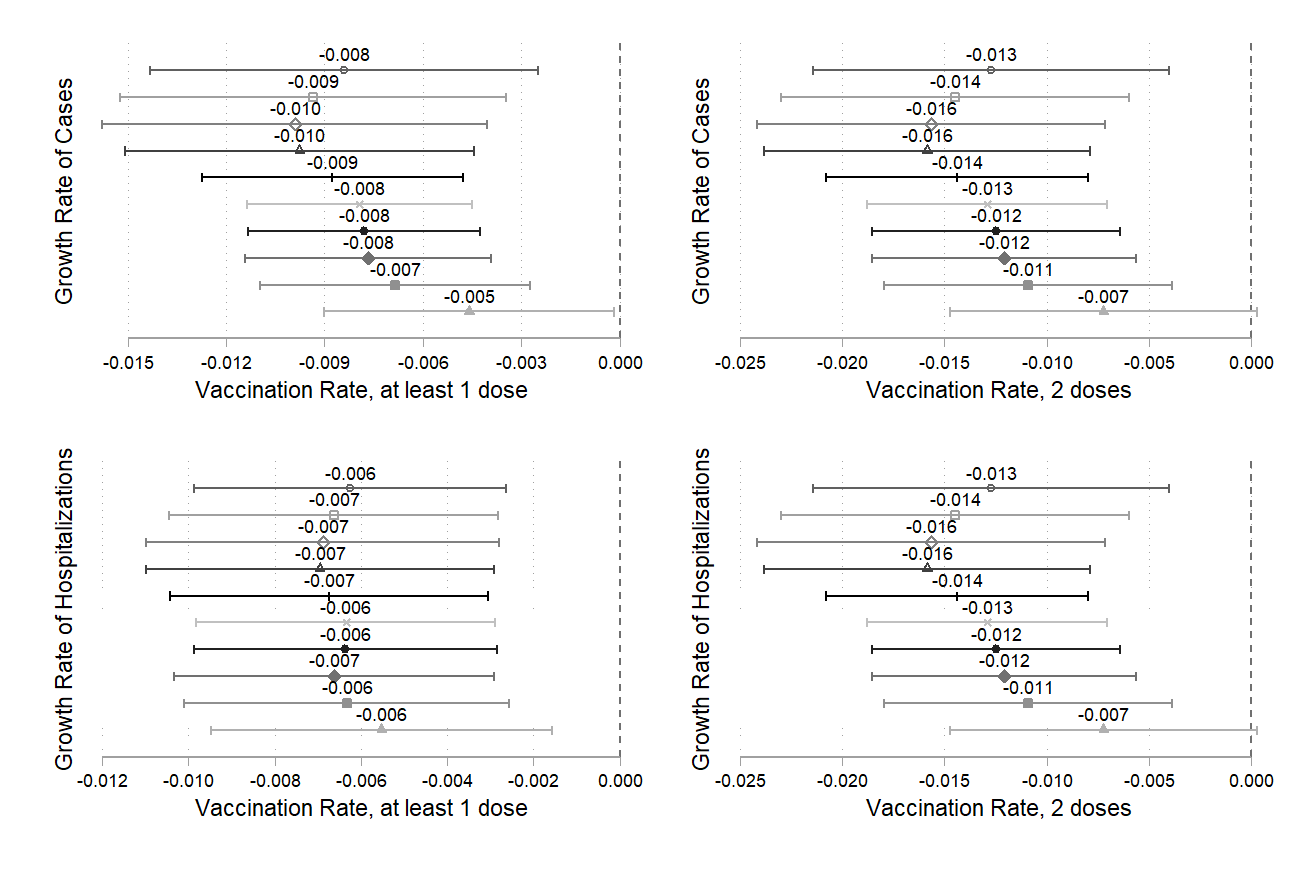

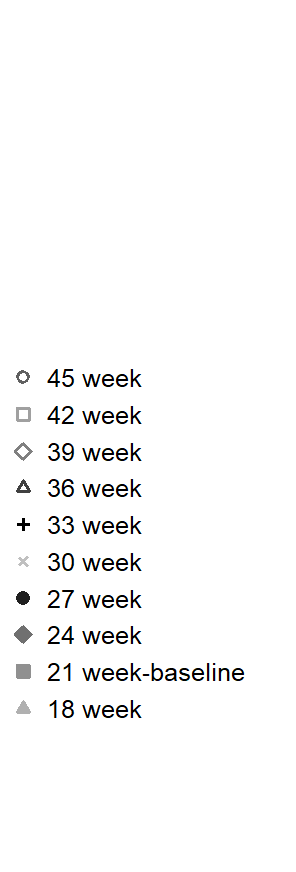


**Extended Data Fig. 6| Estimated effects of vaccination on the COVID-19 pandemic with different sample periods.** Our 21-week baseline period is from 12 October 2020 to 7 March 2021. 18-week period is from 2 November 2020 to 7 March 2021; 24-week from 21 September 2020 to 7 March 2021; 27-week from 31 August 2020 to 7 March 2021; 30-week from 10 August 2020 to 7 March 2021; 33-week from 20 July 2020 to 7 March 2021; 36-week from 29 June 2020 to 7 March 2021; 39 week from 8 June 2020 to 7 March 2021; 42-week from 18 May 2020 to 7 March 2021; and 45-week from 27 April 2020 to 7 March 2021.


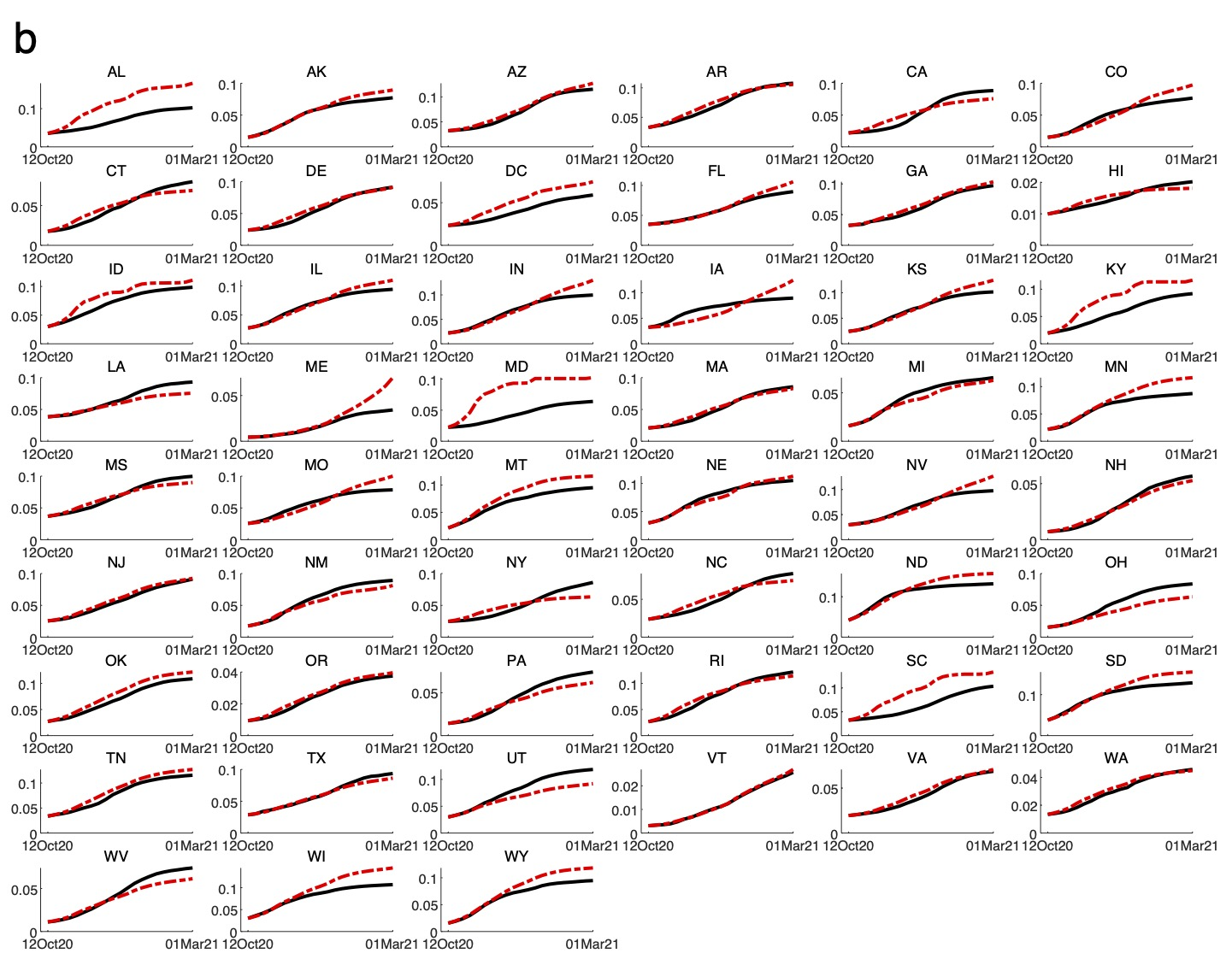


**Extended Data Fig. 7| Development of cumulative infection rate during our study period (12 October 2020 to 7 March 2021).** Red curves are model projections, and black curves are empirical data. **a**, National cumulative infection rate, model projections versus empirical data. Our model projections are 99.69% correlated with empirical data. **b**, Development of cumulative infection rate across all 50 U.S. states and DC, model projections versus empirical data. Our model estimations reached a median correlation of 99.04% with empirical data, with a minimum of 86.37% in Maryland and a maximum of 99.95% in Vermont.


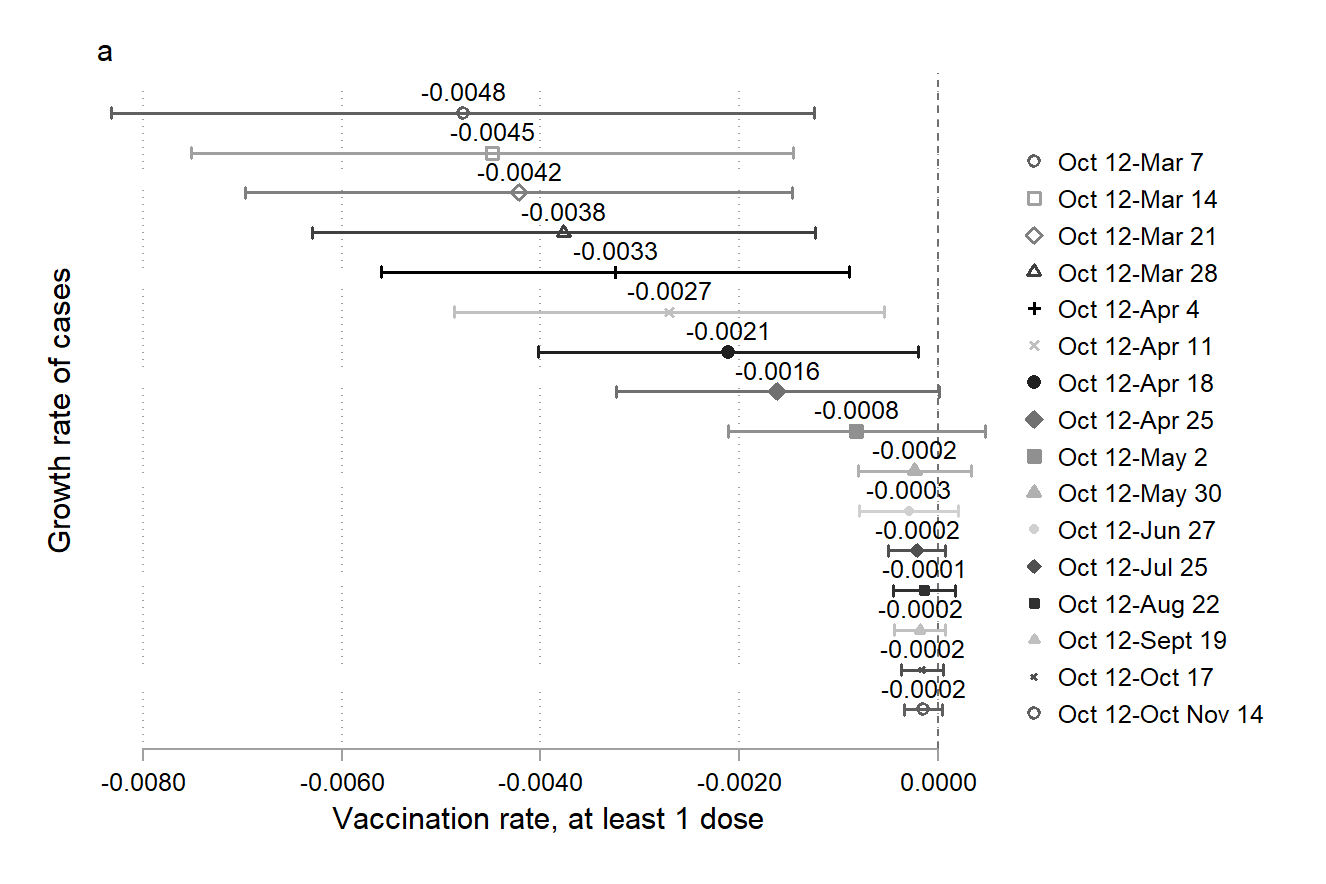

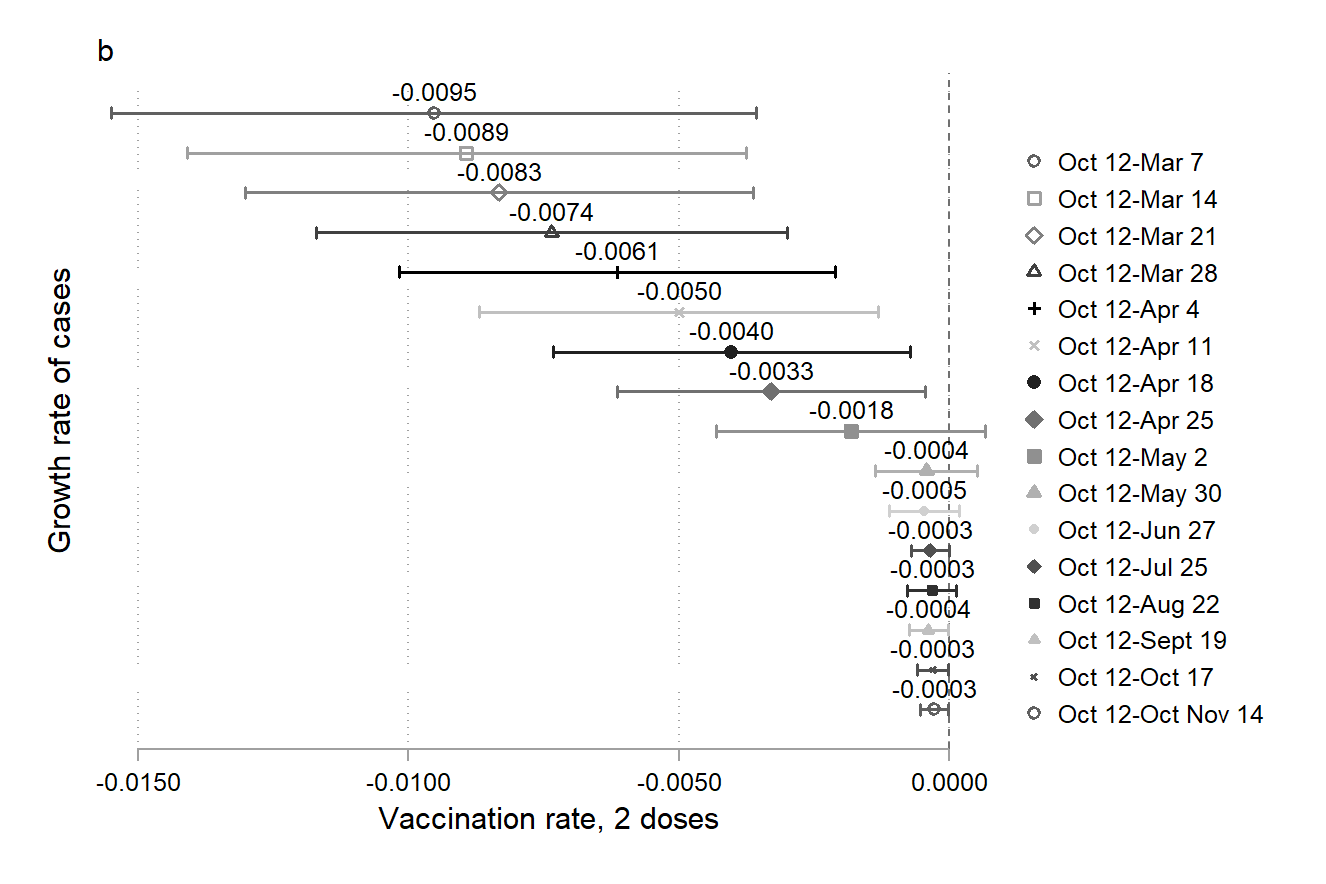


**Extended Data Fig. 8| Estimated effects of vaccination on the COVID-19 pandemic with different sample periods.** Our 21-week baseline period is from 12 October 2020 to 7 March 2021. To provide a more up-to-date estimation, we continue to set the start date at 12 October 2020, and first expand the time window week by week from 7 March to 2 May 2021, and then every four weeks afterward from 30 May to 14 November.


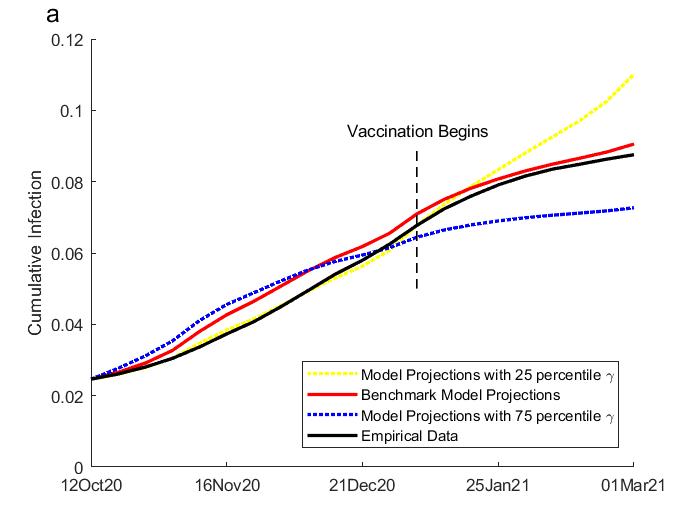


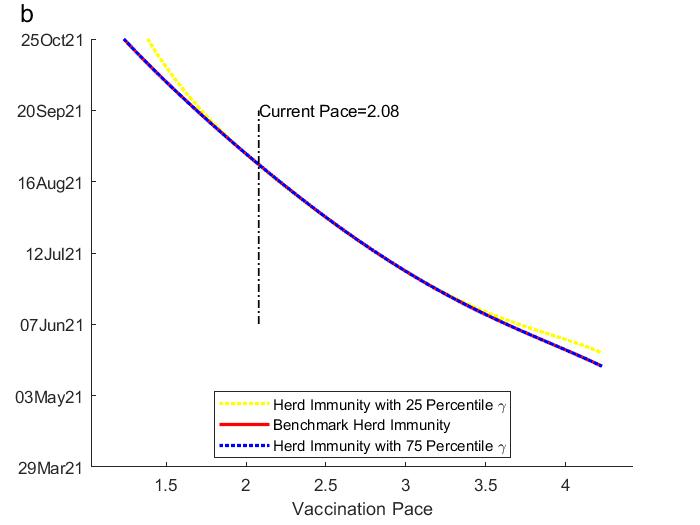


**Extended Data Fig. 9| Model projections on cumulative infection rate and predicted herd immunity date with different imputed removal rate.**

**Extended Data Table 1. Summary statistics.**

| **Variable** | **N** | **Mean** | **SE^a^** | **Median** | **(Min, Max)** |
| --- | --- | --- | --- | --- | --- |
| Growth of total cases | 1071 | 0.07 | 0.05 | 0.06 | (0, 0.3) |
| New cases | 1071 | 19583.4 | 28962.1 | 10544 | (72, 302690) |
| Growth of total hospitalizations | 735 | 0.05 | 0.04 | 0.04 | (0, 0.5) |
| New hospitalizations | 735 | 629.4 | 798.8 | 417 | (5, 13127) |
| Stringency index | 1071 | 46.3 | 13.4 | 46.3 | (7.4, 75.9) |
| People vaccinated per hundred^b^ | 1071 | 4.1 | 6.1 | 0 | (0, 24.7) |
| People fully vaccinated per hundred^c^ | 1071 | 1.7 | 3.0 | 0 | (0, 15.6) |
| Susceptible / population | 1071 | 94.1 | 2.9 | 94.3 | (86.8, 99.7) |
| Testing / population | 1071 | 80.1 | 41.9 | 71.6 | (20.0, 295.4) |
| Rally (dummy variable) | 1071 | 0.03 | 0.2 | 0 | (0, 1) |
| Number of protests | 1071 | 0.06 | 0.4 | 0 | (0, 7) |
| Snow depth | 1071 | 19.5 | 34.0 | 4.5 | (0, 243.3) |
| Deviation from mean temperature^d^ | 1071 | -0.9 | 0.7 | -1.0 | (-3.1, 0.7) |

Notes.

^a^ Standard error.

^b^ People who received at least 1 dose of the vaccine.

^c^ People who received 2 doses of the vaccine.

^d^ Deviation from mean temperature = (temperature at state i week t – mean temp of state i during 12 Oct 2020 to 7 Mar 2021) / standard error of temperature of state i.

**Extended Data Table 2. Baseline regression results.**

| **Variables** | **Growth rate of cases**  (N = 1,071) | | **Growth rate of hospitalizations**  (N = 735) | |
| --- | --- | --- | --- | --- |
| People vaccinated per  hundred^a^ | -0.007*** |  | -0.007*** |  |
|  | (0.002) |  | (0.002) |  |
|  |  |  |  |  |
| People fully vaccinated  per hundred^b^ |  | -0.011*** |  | -0.011*** |
|  |  | (0.004) |  | (0.003) |
|  |  |  |  |  |
| Stringency index | -0.000 | -0.000 | 0.001 | 0.000 |
|  | (0.001) | (0.001) | (0.000) | (0.000) |
| Susceptible rate | 0.021*** | 0.021*** | 0.010*** | 0.010*** |
|  | (0.005) | (0.005) | (0.003) | (0.003) |
| Test rate | 0.000 | 0.000 | 0.000 | 0.000 |
|  | (0.000) | (0.000) | (0.000) | (0.000) |
| Rally | -0.004 | -0.004 | -0.005 | -0.005 |
|  | (0.007) | (0.007) | (0.008) | (0.008) |
| Number of protests | -0.001 | -0.000 | 0.001 | 0.001 |
|  | (0.003) | (0.003) | (0.006) | (0.006) |
| Log (snow depth) | 0.001 | 0.001 | 0.000 | 0.001 |
|  | (0.002) | (0.002) | (0.001) | (0.001) |
| Deviation from mean  temperature^c^ | -0.003 | -0.003 | -0.008 | -0.008 |
|  | (0.006) | (0.006) | (0.006) | (0.006) |
| R-squared | 71.4% | 71.5% | 48.2% | 48.1% |

Notes.
Standard errors (in parentheses) are two-way clustered at state and week level.
Significance levels: *** p<0.01, ** p<0.05, * p<0.1.
^a^ People who received at least 1 dose of the vaccine.
^b^ People who received 2 doses of the vaccine.
^c^ 21 observations of DC temperature were missing, estimated using average temperature from neighboring states of Virginia and Maryland. 8 observations of Delaware temperature were missing, estimated using average temperature from the neighboring states of New Jersey and Maryland.

**Extended Data Table 3. Baseline regression results with data of daily frequency.**

| **Variables** | **Growth rate of cases**  (N = 7,497) | | **Growth rate of hospitalizations**  (N = 5,145) | |
| --- | --- | --- | --- | --- |
| People vaccinated per  hundred^a^ | -0.0009*** |  | -0.0009*** |  |
|  | (0.0002) |  | (0.0002) |  |
|  |  |  |  |  |
| People fully vaccinated  per hundred^b^ |  | -0.0015*** |  | -0.0015*** |
|  |  | (0.0003) |  | (0.0003) |
|  |  |  |  |  |
| Stringency index | 0.0001 | 0.0001 | 0.0001* | 0.0001 |
|  | (0.0001) | (0.0001) | (0.0001) | (0.0001) |
| Susceptible rate | 0.0027*** | 0.0027*** | 0.0012*** | 0.0013*** |
|  | (0.0005) | (0.0005) | (0.0003) | (0.0003) |
| Test rate | 0.0000 | 0.0000 | 0.0000 | 0.0000 |
|  | (0.0000) | (0.0000) | (0.0000) | (0.0000) |
| Rally | -0.0002 | -0.0002 | -0.0007 | -0.0007 |
|  | (0.0013) | (0.0013) | (0.0016) | (0.0016) |
| Number of protests | -0.0000 | 0.0000 | -0.0007 | -0.0007 |
|  | (0.0009) | (0.0010) | (0.0008) | (0.0008) |
| Log (snow depth) | -0.0002 | -0.0002 | -0.0000 | 0.0000 |
|  | (0.0002) | (0.0002) | (0.0002) | (0.0002) |
| Deviation from mean  temperature^c^ | -0.0007* | -0.0007* | -0.0004 | -0.0004 |
|  | (0.0004) | (0.0004) | (0.0004) | (0.0004) |
| R-squared | 51.7% | 51.8% | 16.4% | 16.4% |

Notes.

Standard errors (in parentheses) are two-way clustered at state and week level.
Significance levels: *** p<0.01, ** p<0.05, * p<0.1.

^a^ People who received at least 1 dose of the vaccine.
^b^ People who received 2 doses of the vaccine.
^c^ 21 observations of DC temperature were missing, estimated using average temperature from neighboring states of Virginia and Maryland. 8 observations of Delaware temperature were missing, estimated using average temperature from the neighboring states of New Jersey and Maryland.

**Extended Data Table 4**. **Predicted herd immunity date over possible infection and removal rates**, **evaluating the impact of the Delta variant.**

| Infection Rate | | 120% | 140% | 160% | 180% | 200% |
| --- | --- | --- | --- | --- | --- | --- |
| **Removal Rate** | **60%** | 27 Jun 22 | 02 May 22 | 21 Mar 22 | 17 Jan 22 | 29 Nov 21 |
|  | **70%** | 20 Jun 22 | 25 Apr 22 | 14 Mar 22 | 07 Feb 22 | 03 Jan 22 |
|  | **80%** | 31 Jan 22 | 18 Apr 22 | 07 Mar 22 | 31 Jan 22 | 03 Jan 22 |
|  | **90%** | 17 Jan 22 | 14 Mar 22 | 28 Feb 22 | 24 Jan 22 | 27 Dec 21 |
|  | **100%** | 27 Dec 21 | 07 Mar 22 | 07 Mar 22 | 24 Jan 22 | 27 Dec 21 |

Notes.

This table shows how the predicted herd immunity date varies over a range of possible values of the infection rate and removal rate (recovery rate + death rate) using the our baseline SIR model. The values of the infection rate and removal rate reflect estimates for the Delta variant, compared with previous variants.
